# Rejection of *Lepeophtheirus salmonis* driven in part by chitin sensing is not impacted by seawater acclimitization in Coho salmon (*Oncorhynchus kisutch*)

**DOI:** 10.1038/s41598-023-36632-0

**Published:** 2023-06-15

**Authors:** Laura M. Braden, Dylan Michaud, David Groman, Phil Byrne, Tiago S. Hori, Mark D. Fast

**Affiliations:** 1 Department of Pathology and Microbiology, Atlantic Veterinary College, Charlottetown, PE Canada; 2 Department of Fish Health and Molecular Biology, AquaBounty Canada, Souris, PE Canada; 3 Aquatic Diagnostic Services, Atlantic Veterinary College, Charlottetown, PE Canada; 4 Department of Fisheries and Oceans Canada, Charlottetown, PE Canada; 5 Atlantic Aqua Farms, Borden, PE Canada

**Keywords:** Transcriptomics, Parasitic infection, Acute inflammation, Gene expression profiling, Immune evasion, Dendritic cells, Pattern recognition receptors, B cells, NK cells, T cells, Mucosal immunology

## Abstract

There is tremendous variation in life-history strategies among anadromous salmonids. Species that enter the ocean environment at small sizes (< 20 g) are likely under more physiological pressure from pathogens; however, little data is available on responses at these early stages. With this in mind, we performed salmon louse challenges with Coho salmon either immediately after seawater entry (SW; ca. 10 g) or after 30 days in SW (ca. 20 g). Irrespective of size or time in SW, parasites were rapidly rejected by the host, with > 90% of all parasites lost by 16 days post-infection (dpi). Rejection was concomitant with host epithelial granulomatous infiltrations that initially targeted the embedded frontal filament (4 dpi) and the entire parasite by 10 dpi. Illumina sequencing, followed by functional enrichment analysis, revealed a concerted defense response in the fin within 1 dpi that included multiple innate and adaptive immunity components. Strikingly, early indications of an allergic-type inflammatory response were associated with chitin sensing pathways orchestrated by early overexpression of the IgE-receptor, *fcer1g*. Additionally, there was profound overexpression of several classes of c-type lectin receptors, including *dectin-2*, *mincle*, and *dc-sign* at 1 dpi onward. These profiles and upregulation of cellular effector markers were corroborated by histopathological evaluation, revealing the simultaneous presence of mast cell/eosinophilic granular cells, sacciform cells, macrophages/histiocytes, and granulocytes in fin. At 10 dpi and concurrent with parasite expulsion, there was evidence of immunoregulation in addition to tissue remodelling pathways. At 16 dpi, the response was effectively abrogated. Simultaneous profiling of the parasite transcriptome revealed early induction of chitin metabolism and immunomodulation, toxin production and ECM degradation; however, after 7 dpi, these were replaced with overexpression of stress and immune defense genes. These data present the first evidence for Coho salmon demonstrating chitin- and sugar moiety-sensing as key drivers of salmon louse rejection.

## Introduction

Anadromous salmonids emerge as yolk-sac fry in fresh water streams and lakes but soon migrate to the marine environment to feed and grow before returning to their natal streams to spawn, and most often, die^1^. This evolutionary life-history adaptation has been associated with differences in resource availability. The benefit of increased productivity in the marine environment and greater fecundity due to larger body size significantly outweigh the costs of increased mortality due to smoltification and predation^2^. In the Pacific Ocean, there has been a considerable evolutionary divergence of the salmonids from a common ancestor ca. 15–20 Mya, resulting in five species of salmon. Each species has a distinct life history, physiology, and behavior^3,4^. For example, despite similar biogeographic ranges, Pacific salmonids are spatially segregated within the same watershed^3,4^. These differences are observed in general behavior throughout development, including the size of seawater entry^2,4^. During the shift from freshwater (i.e., parr) to marine habitat (i.e., smolts), there is tremendous physiological stress on salmonids as they must transition to survive in seawater, including changing morphology, increasing metabolic rate, ion regulation, and immunity^3–5^. Particularly important to survival during smoltification is being equipped to withstand the pathogenic onslaught by novel pathogens present in the marine habitat. This might be expected to be critical for salmon that enter the ocean at smaller sizes than others, such as Pink *Oncorhynchus gorbuscha* and Chum salmon *O. keta*, which enter the marine environment shortly after emerging as fry (ca. 0.5 g;^3,4^).

Ectoparasitic salmon lice *Lepeophtheirus salmonis* (*Lsal*) are considered as one of the most significant constraints to the sustainable expansion of commercial salmon aquaculture in the Northern hemisphere^6^. Accounting for over US $1 billion in losses to the industry annually, *Lsal* causes epidermal erosion, chronic wounds, osmoregulatory distress, secondary infections, and concomitant losses to production (reviewed in 6). These effects are, however, only associated with certain host species as a variable host susceptibility occurs among the salmonidae^7^. For example, Pink and Coho (*O. kisutch*) salmon exhibit resistance as juveniles, with the acquisition of resistance in Pink salmon occurring at around 0.7 g^8^. Planktonic (copepodite stage) sea lice have limited energy reserves and upon encountering a host they must acquire the appropriate nutrition to further penetrate the host epithelium, moult (to Chalimus I stage) and extrude a frontal filament to maintain attachment to survive. Resistant hosts have the ability to arrest/prevent the development of parasites from the attached chalimus stages to adulthood. Differential susceptibility has been of extreme interest to research programs, as understanding what drives parasite rejection in some species would be important to the industry.

Control of *Lsal* has primarily been accomplished through the application of chemotherapeutics. However, the over-use of a limited repertoire of compounds is associated with the development of resistance in multiple farming regions^9^. Several groups have attempted to develop a vaccine against *Lsal* with limited success, reporting a maximum of 29% reduction in adult female abundance and in some cases reduced fecundity^10,11^. In the absence of an efficacious vaccine or substantial gains in resistance through selective breeding, novel control strategies are urgently needed. Understanding molecular mechanisms driving host resistance observed in Pink and Coho salmon may represent such an opportunity, as these mechanisms are likely applicable to Atlantic salmon through careful, targeted selection measures, application of therapeutics to target identified pathways, or precision editing technologies.

With respect to Coho salmon, the molecular mechanisms driving the profound rejection of *Lsal* have remained enigmatic. The first description of the rejection response demonstrated an aggressive epithelial hyperplasia and recruitment of cellular effectors that was effectively abrogated upon exposure to corticosteroids^12,13^. This host response has been since confirmed to be specific to Coho salmon, and rejection found to occur across both sub-species of *L. salmonis* and to *Caligus rogercresseyi* from farmed Coho salmon in Chile. Subsequent studies focused on enumerating certain genes found to be dysregulated in the response by susceptible species resulting in hypotheses related to nutritional immunity^8^, aberrant wound healing and inflammatory cascades^14,15^ and enhanced parasite immunomodulation^16,17^. Furthermore, as discussed above, there is a developmental contribution, as lice resistance in Pink salmon is only observed at juvenile stages^18,19^.

In the present study, we sought to identify mechanisms involved during the rapid rejection of salmon lice by its resistant host species, Coho salmon. We also wanted to determine, if similar to Pink salmon, differences exist in coho salmon based on size at exposure. To achieve this goal, we infected post-smolt Coho (at two sizes/sea water acclimatization) with infective *Lsal* copepodites and used whole transcriptome RNA-sequencing to profile both host and parasite transcriptomes (dual RNA-seq) as the infection progressed from 24 h and throughout the period of host rejection. Dual RNA-seq allows for the simultaneous analysis of transcriptomes between two or more interacting partners, and this approach has been applied to investigate a variety of commensal, mutualistic, and parasitic relationships^20^. For example, dual transcriptome profiling during pathogen infections has permitted the identification of important regulators of the host-parasite interaction, including important hubs of virulence responsible for disabling host immunity^21,22^. Here we describe the first host-parasite interactome which illuminates critical pathways in the resistance phenotype that results in hyperplastic encapsulation and subsequent killing of attached *Lsal*.

## Results/discussion

### Salmon louse rejection by Coho salmon is independent of time in seawater or host size

**Figure 1:**
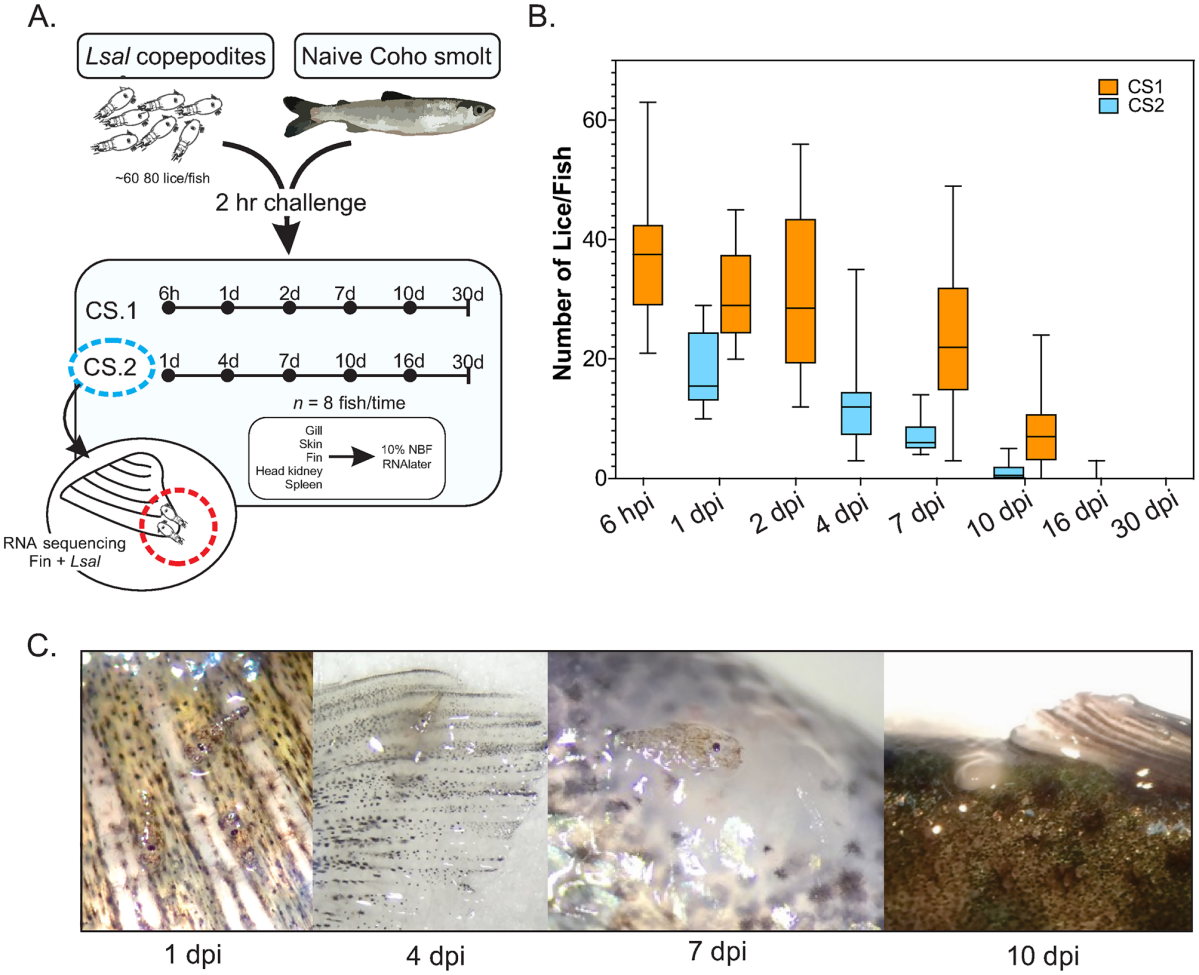
Experimental salmon lice challenge of Coho salmon. (**A**) Naïve *Okis* were exposed to infective *Lsal* copepodites over two separate trials; CS1, where *Okis* were in full saltwater for ~ 24 h, or CS2, where *Okis* were in full saltwater for ~ 30 days. Coho salmon were sampled and fin with attached *Lsal* were preserved for RNA sequencing. (**B**) Coho salmon from both trials rapidly rejected *Lsal*, with > 95% of lice lost by 16 dpi. (**C**) Epithelial hyperplasia and granuloma formation were observed associated with the anterior portion of the lice by 4 dpi. By 10 dpi the lice were most often completely encapsulated. Except for 3 pre-adult mobile lice, there we no lice observed at 16 dpi.

Resistance to *Lsal* has been associated with size in juvenile salmon, with Pink salmon achieving resistance after surpassing 0.7 g, coincident with thickening of the epidermis and development of scales^8,18^. Determination of size-specific associations with resistance have never been performed in Coho salmon. We conducted an experimental challenge where 1-day (CS1: weight = 11.7 ± 3.6 g, fork length = 10.4 ± 1.3 cm) or 30-day (CS2: weight = 18.6 ± 4.9 g, fork length = 12.1 ± 0.9 cm) Coho salmon smolts, *O. kisutch (Okis)*, were exposed to ~ 60–80 copepodites fish^−1^ (Fig. 1A) to investigate the hypothesis that this species would have greater resistance to salmon lice after longer acclimatization to sea-water (TIS).

Although the dynamics of parasite rejection were slightly variable between the two challenges, there was a significant decrease in parasite abundance over time irrespective of size or TIS, with most lice rejected by 16–18 dpi. Attachment of parasites in CS2 may have been reduced compared to CS1 as the 1 dpi time point was significantly lower in CS2, which may have been due to some variation in parasite robustness. Despite the initial difference both infections trended similarly, suggesting the TIS had no impact (Fig. 1B). Concomitant with reductions in *Lsal* abundance, we observed the formation of granulomatous infiltrate within the fin and skin of infected salmon as early as 4 dpi, again independent of size or TIS (Fig. 1C). In both challenges, epithelial hyperplasia and granulomatous infiltrate was associated with the anterior end of the attached larvae, surrounding the embedded frontal filament and concurrent with *Lsal* molt to chalimus I. Granulomas are aggregations of macrophages and other immune cells that form at sites of chronic inflammation or around foreign bodies, and as the infection progressed, the granuloma grew to encase the attached parasite. By 10 dpi, the cellular response presented grossly as an opaque scar and the parasite was no longer evident (Fig. 1C). At 16 dpi, we no longer observed granulomas, and the total number of parasites that remained after both trials were 3 mobile pre-adults in total. This supports earlier observations in the laboratory and the field, where only mobile *Lsal* are observed on Coho salmon^23,24^.

**Figure 2:**
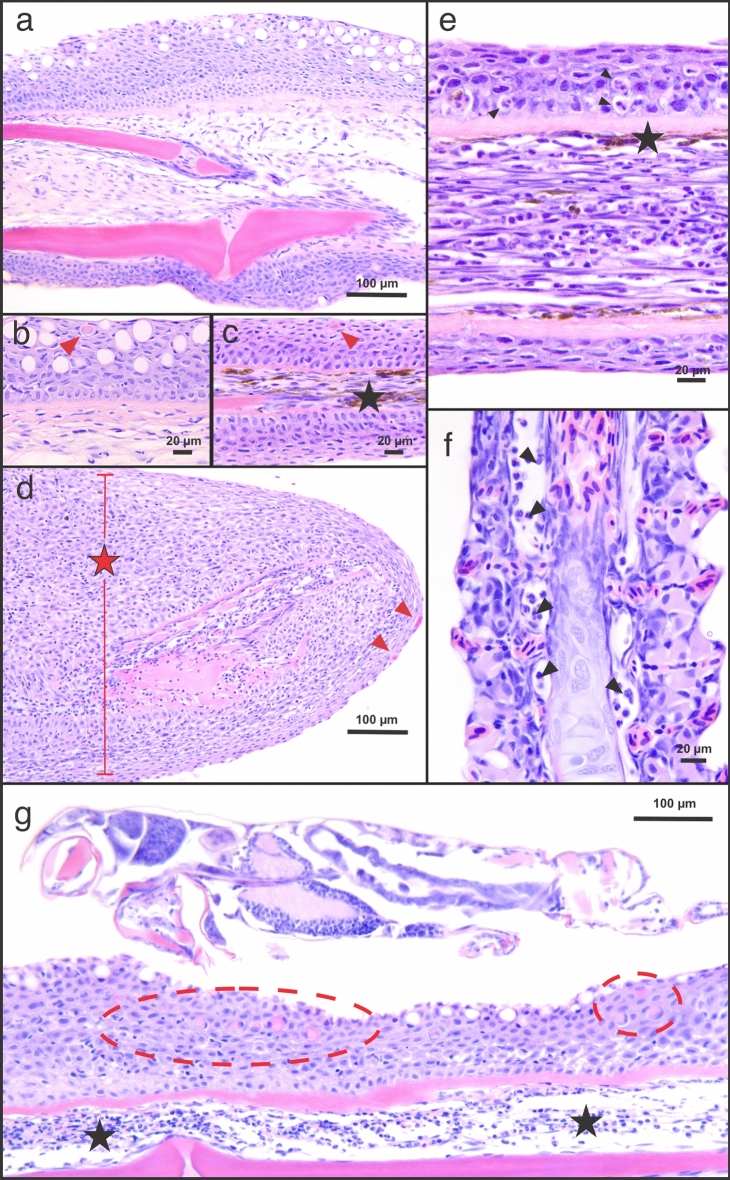
Cellular response of Coho epithelium to salmon lice. Representative histopathology of *Okis* fin and gills during the host response 4–7 dpi revealed several populations of cells involved in the epithelial response to *Lsal* (**a**–**g**). Whereas control fin was characterized by populations of mucocytes (**a**, **b**), infected fin was characterized by thickened epithelium and mucocyte hypoplasia (**c**–**d**). Sacciform cells (red arrowheads) were present in control and infected fin and were typically associated with the apical surface, however, they were more abundant at the louse attachment sites (red circles, **g**). Accumulation of melanomacrophages was evident in infected fin (**c**, **e**; star), and this was often associated with leukocytes as observed in fin and gill (**e**–**f**; black arrows). Epithelial hyperplasia was evident by 4 dpi (**d**, red star), and this response included significant presence of lymphocytes (**f**, **g**), and granulomatous inflammation (**g**; star).

Histopathological assessment of infected fins revealed moderate infiltration of granulocytes, including neutrophils, leukocytes, and lymphocytes in mucosal epithelia as early as 1 dpi (Fig. 2). Sacciform cells, a cell type found in the epithelium of certain salmonid species and are thought to possess anti-parasitic activity^25^, were observed throughout the infestation. At times they also appeared to be associated with the louse attachment site (Fig. 2b–d). Furthermore, mucocyte hypoplasia was observed in *Lsal*-infected fin as early as 1 dpi. Infiltration of cellular effectors increased by 4 dpi, where significant melanin deposition was observed in the fin and evidence of epidermal hyperplasia was present (Fig. 2c,e).

By 7 dpi, granulocyte infiltration was reduced, and epidermal hyperplasia intensified (Fig. 2d). Eosinophilic granular cell/mast cell (referred to as MCs for brevity) infiltration was observed in the gill arch and fin subcutis throughout the infection. These cells were associated with the attached parasite at 4 dpi, and MCs were often associated with neutrophils and lymphocytes (Fig. 2e–g).

### Characterization of the host-parasite transcriptomes

To understand the molecular pathways driving parasite rejection in Coho salmon, we used RNA-sequencing to characterize transcriptome changes in *Lsal*-infected fin compared to uninfected fin samples from the second exposure experiment (CS2; *n* = 35 fish). Using a dual RNA-seq approach, we simultaneously evaluated the responses in both host and parasite throughout the infection from 1 to 16 dpi. A total of ~ 727 million paired-end 125 bp reads were generated. These were adapter trimmed and low-quality read filtered, before the trimmed data were deposited on the SRA database (BioProject ID PRJNA765642).

All high-quality, trimmed reads were first mapped to the RefSeq *O. kisutch* assembly (GCF 002,021,735). Unmapped reads were extracted from BAM files and aligned to the *Lepeophtheirus salmonis* assembly (ASM18125v2). Of the 651,763,086 high-quality reads obtained from uninfected and infected fins, 82.39–91.72% were of *Okis* origin, 0.98–5.42% were of *Lsal* origin, while unmapped reads varied from 7.30 to 12.19% (Suppl. Fig. 1). In the controls, 0.03% of reads from uninfected controls contained mapped to the *Lsal* RNA, likely due to mapping errors, and many samples at both 1 and 16 dpi contained low *Lsal* transcript abundance. This result can be attributed both to the challenge of sampling attached lice before frontal filament extrusion at 1 dpi and the almost complete parasite rejection by 16 dpi, respectively (S1 Table).

Parasite RNA increased over time, peaking at 10 dpi, reflecting the growth of attached *Lsal* from copepodite through to chalimus. After 10 dpi there was a decrease in *Lsal* transcripts, as the host rejected the parasite. Principal component analysis of transcriptional profiles supported this pattern, where infected samples at 4, 7, and 10 dpi clustered together, while non-infected controls, 1 dpi and 16 dpi, clustered more closely with controls (Supp. Fig. 1).

Evaluation of the individual samples associated with these two sub-clusters revealed a contribution of *Lsal* RNA abundance being associated with host transcriptional responses (Supp. Fig. 1). The impact of variable pathogen/parasite abundance in driving host responses has been demonstrated before^26^. In this study, sub-cluster 2a contained the samples with the highest percent *Lsal* reads with 6.13 ± 3.61%, while sub-cluster 2b had an average *Lsal* read count of 3.55 ± 3.48%. Samples in cluster 1, composed of 1 dpi, 16 dpi, and controls, had an average *Lsal* read count of 0.35 ± 0.53%, significantly lower than either of the subclusters. Although the difference in the % parasite reads between *Lsal*-low and *Lsal*-high clusters was not significant (*p* = 0.094), there was a clear trend of parasite RNA being associated with the clustering of samples from 4, 7 and 10 dpi.

**Figure 3:**
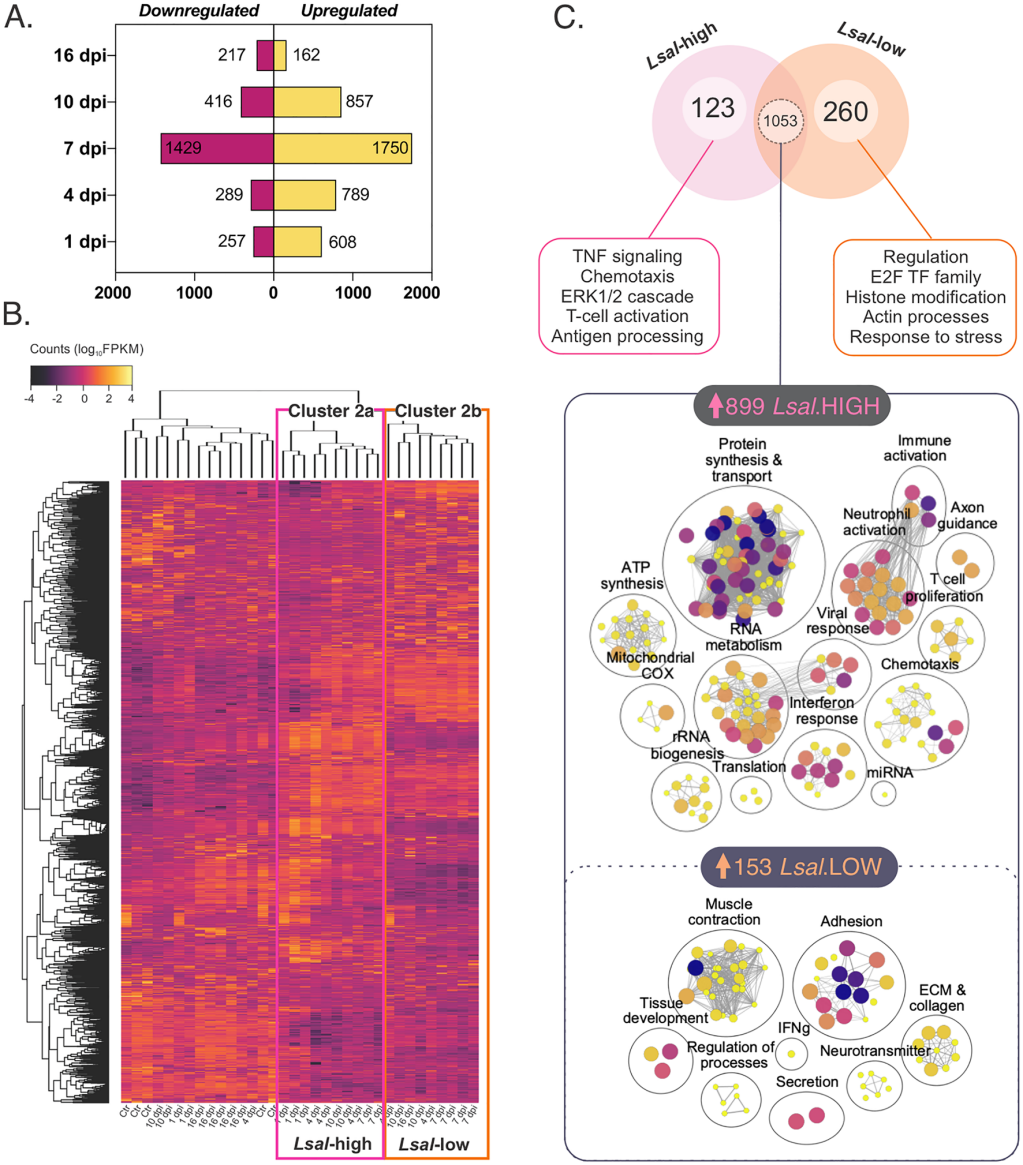
Differential expression analysis of transcripts using DEseq2. (**A**) The number of DETs upregulated (yellow) or downregulated (pink) in *Okis* fin, compared to the 1dpi reference control, throughout the infection period (with an adjusted *p*-value of < 0.01). (**B**) Within these DETs, hierarchical expression profiling detected a divergence (three main clusters of samples; 1, 2a, 2b) in the infected samples that correlated with the presence of *Lsal* reads. (**C**) Taking the clusters making up 2a and 2b and treating them as *Lsal*-high or *Lsal*-low groups revealed that these two clusters involved differing transcriptomic profiles (123 DETs unique to *Lsal*-high and 260 DETs unique to *Lsal*-low; 1053 shared DETs of which 899 were higher expressed in *Lsal*-high and 153 of which were more highly expressed in *Lsal*-low).

Differential expression analysis by DESeq2 identified 2984 host and 573 parasite transcripts that were significantly induced or repressed (DETs; FDR < 1%, Fig. 3A, S1 File) in *Lsal*-infected fin associated with the rejection response over time. Hierarchical clustering and PCA demonstrated that the apex of the host response occurred at 7 dpi, as evidenced by the most significant number of distinct DETs (Fig. 3B). By 16 dpi, the response was largely abrogated, concomitant with complete rejection of parasite by that time (Fig. 1B). From 4 to 10 dpi, the fin response was the most homogenous in the number of shared DETs, again characterized by a divergence that was significantly correlated with *Lsal* mRNA. Detected changes in the abundance of DETs in Coho fin reflected expression changes in the fin but also variations in the cell composition of the fin due to infiltrating cells (Fig. 2). Estimates of fold changes by DESeq2 of select host transcripts were confirmed by quantitative RT-PCR (S2 File).

### Variability in host response dynamics does not impact rejection

To explore the genes responsible for driving the divergent profiles within the two subclusters (*Lsal*-high and *Lsal*-low) in infected fin, we further evaluated transcripts that were differentially expressed (log2 FC >|1|, coefficient of variation < 1.5) between them (S3 File). There were 123 DETs specifically upregulated in the *Lsal*-high group and 267 transcripts specific to the *Lsal*-low group, while 1053 DETs were differentially expressed between *Lsal*-low and *Lsal*-high groups (Fig. 3C).

Further analysis revealed the *Lsal*-low cluster was characterized by overexpression of genes involved in positive regulation of many processes associated with homeostasis and regulation, which was accompanied by significant enrichment of transcription factors in the E2F family. Upregulation in several immune regulatory molecules in the *Lsal*-low cluster, including inhibitors of cytotoxic T lymphocyte (CTL) killing and T-cell proliferation (*ceacam1*), collagens (e.g., *col10a1, col17a1*), cellular adhesions (e.g., *kcam, dsg2*), and other late-phase wound healing genes (Fig. 3C), would suggest this cluster is associated with the resolution phase of infection. In contrast, DETs specifically enriched in *Lsal*-high samples were associated with TNF signalling, chemotaxis, and T cell activation (Fig. 3C). In the genes differentially expressed between the two clusters, energy production (e.g., *cycs*, *cox4i1*, *cox4i2*), inflammation (e.g., *casp1, il12b, il17d, il18, il1r2*), cellular recruitment (e.g., *il8, ccl2, ccl10*), and complement (e.g., *c5ar1, cfd, cfh*) were upregulated in *Lsal*-high. Cellular recruitment and activation would be suggestive of an active response to the parasite (i.e. not yet resolved) Variable host profiles among individuals at the same time is likely a consequence of variation in parasite intermoult phase paired with variable host response dynamics.

### Coho rejection responses peak at 7 dpi

**Figure 4:**
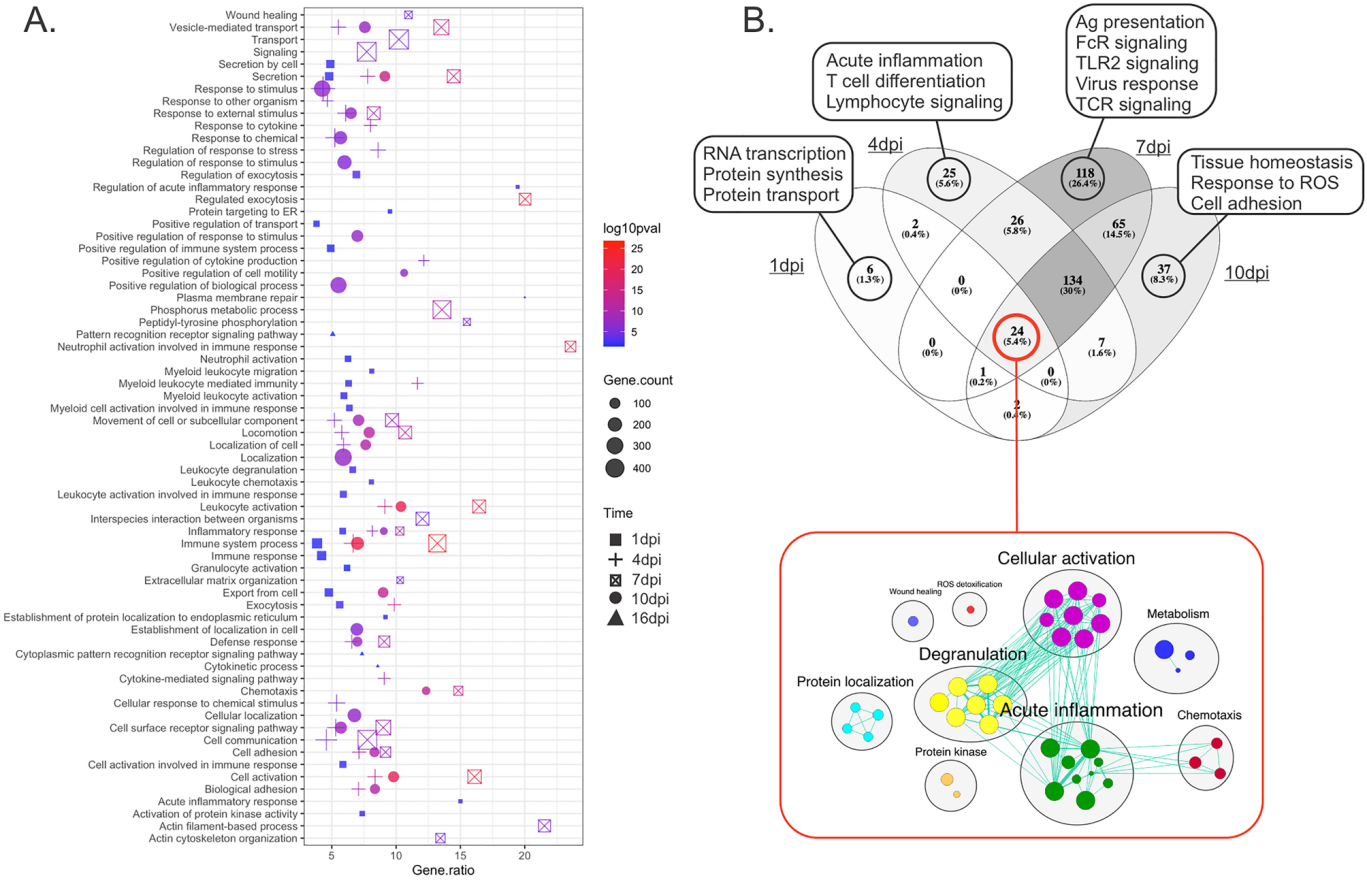
Pathway term enrichment of DETs in the fin transcriptome of *Okis*. (**A**) The top 40 GO categories enriched over time are represented by a bubble plot, with the color denoting significance and size denoting the numbers of genes in each category. (**B**) Comparative analysis of enriched terms at 1, 4, 7 and 10 dpi demonstrated patterns specific to each time point and commonly enriched terms throughout the response. Percentages in brackets describe how the individual DEGs relate to the percentage of total differentially expressed genes. The inset network below, refers to the functions of the 24 DEGs shared across all time points.

To identify biological processes, regulators, and pathways involved during infection with *Lsal*, annotated DETs associated at each time point were analyzed by functional enrichment analysis. Whole transcriptome analysis relying solely on identifying enriched pathways using differentially expressed transcripts may underestimate important regulators or pathways in datasets, particularly when limiting to significant fold-change differences^27^. Pairing functional enrichment of DETs and GSEA together enabled a comprehensive description of processes and pathways involved in parasite rejection. The Coho fin response was initiated early being dominated with signatures of energy metabolism, RNA processing, protein synthesis, and activation of cellular effectors, changing dramatically to involve innate immunity, chemotaxis, cellular recruitment pathways, and extracellular matrix reorganization by 4–7 dpi, when the peak transcriptome change was observed (Figs. 3A and 4A, S2 table). After 10 dpi, the enrichment signatures again changed and reflected resolution of inflammation, wound healing, and return to cellular homeostasis.

Common fin response signatures throughout the infection (1 10 dpi) included degranulation, regulation of wound healing, acute inflammation, and activation of cellular effectors (Fig. 4B; Supp. Fig. 2). Interestingly, at 4 dpi and simultaneous with evidence of granuloma formation and frontal filament extrusion by *Lsal*, there was enrichment in ‘TNF signalling’ (KEGG:04,668), ‘CLR signalling’ (KEGG:04,625), ‘Neutrophil degranulation’ (REAC:R-HSA-6798695), ‘Signalling by interleukins’ (REAC:R-HSA-449147), ‘IL-4/13 signalling’ (REAC:R-HSA-6785807), and ‘Arachidonic acid metabolism’ (REAC:R-HSA-2142753) pathways (Supp. Fig. 2; S3 Table). Investigation of the genes associated with these pathways revealed several overlapping DETs, including hallmark inflammatory mediators *il1*b, *tnf*a, *il12b*, cell signalling regulators *jak1, jak2*, *cebpb*, and *socs3,* and chemokines *cxcl8* and *ccl2* (Supp. Fig. 2). At 10 dpi, enriched pathways were associated with ECM remodeling and tissue homeostasis, while at 16 dpi the only enriched pathways were ‘Endocytosis’ (KEGG: 04,144) and ‘Dectin-2 family’ (REAC:R-HSA-5621480).

### Tissue-resident mast cell- and macrophage-signaling drive salmon lice responses in Coho salmon

**Figure 5:**
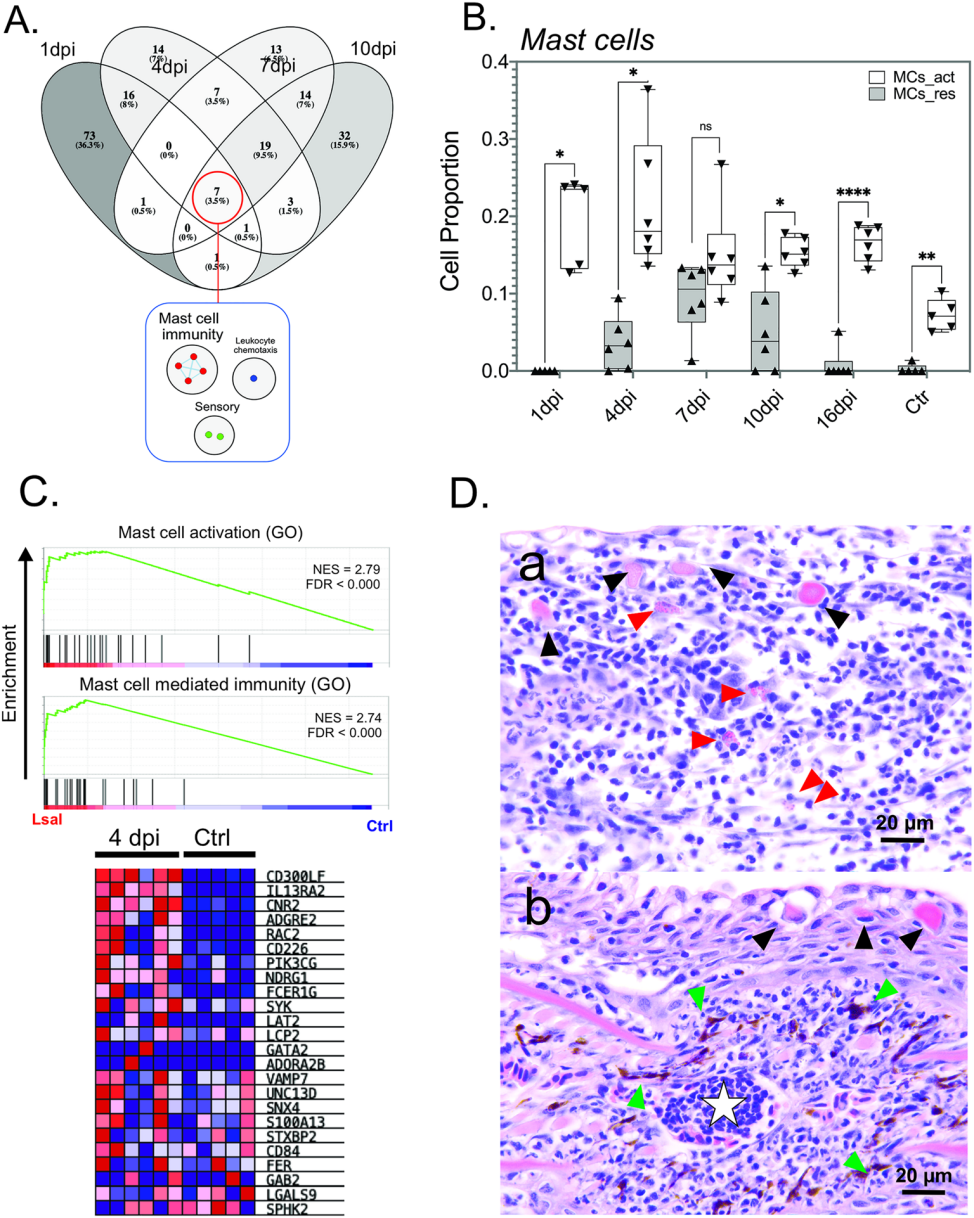
GSEA detected a significant enrichment of mast cell response pathways in the fin transcriptome of *Okis*. (**A**) VENN analysis of significantly enriched GO categories using GSEA from every timepoint indicated that mast cell immunity was common throughout infection. (**B**) Activated and resting MCs were detected in the transcriptome and were significantly higher than controls by 4–7 dpi. (**C**) Enrichment plot from GSEA of two associated GO categories showing the normalized enrichment score (NES) and FDR. Expression profiles of the major genes are shown as a heatmap from the 4-dpi dataset. (**D**) Histopathology of the mucosal epithelium (gill, (**a**); fin (**b**)) at 4 dpi showing MCs (red arrows), sacciform cells (black arrows), melanomacrophage centres (green arrows), and accumulating lymphocytes (white star).

Allergic inflammation is activated in response to allergens associated with macroparasites, such as toxins, hematophagous fluids, and chitin-associated molecules^28^. Potential for allergenic inflammation to Lsal occurs at settlement and attachment as exoskeletal (composed of chitin and other immunogenic compounds) and virulence-associated molecules penetrate the physical barrier of the skin epithelium. The GSEA conducted here suggests a dominant role for mast cells, the main cellular drivers of allergic inflammation^29^, in potentiating louse-rejection by Coho salmon as mast cell-related functional enrichment was significant throughout the infection (Fig. 5).

**Figure 6:**
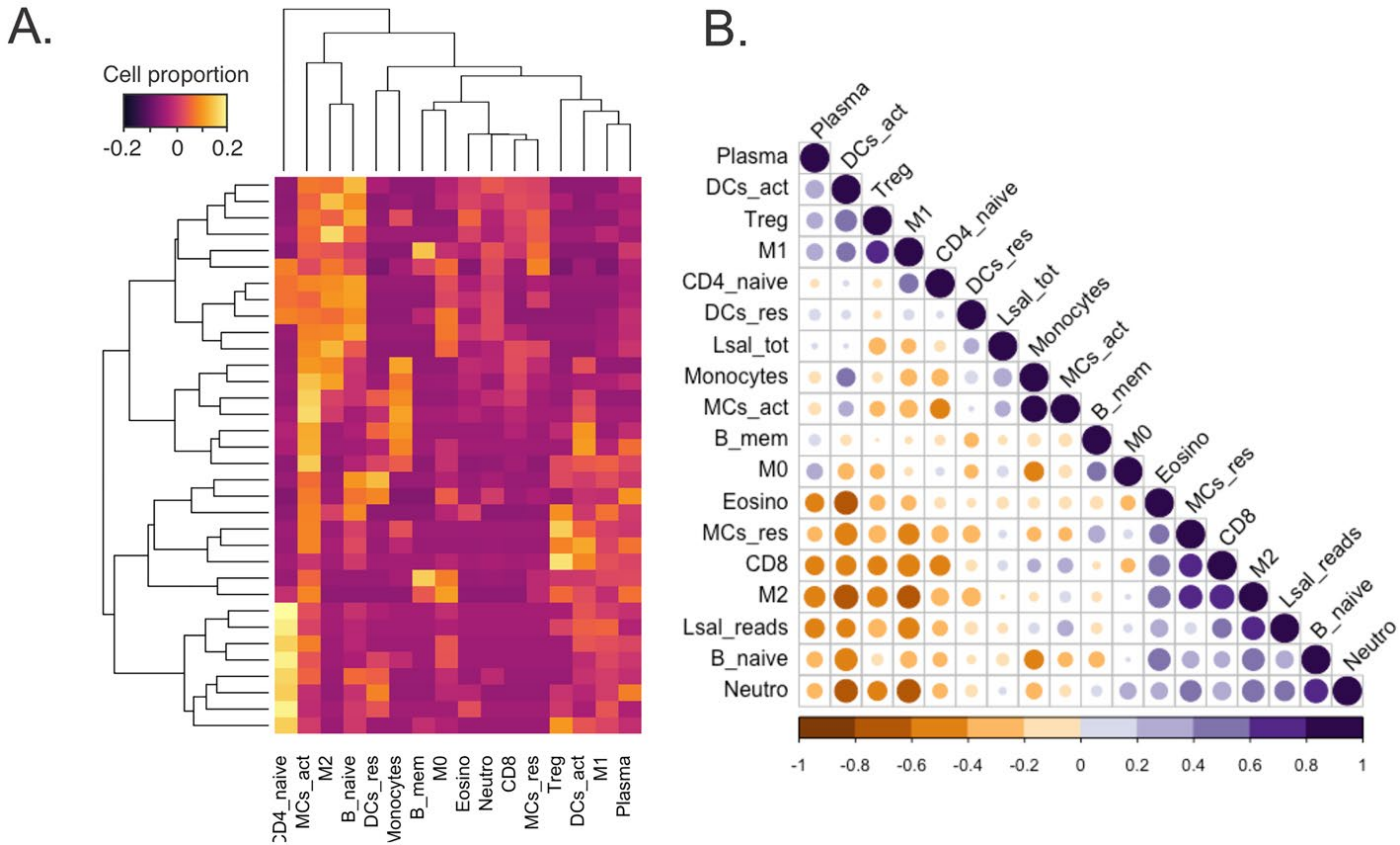
Proportions of cellular effectors were detected in the fin transcriptome by deconvolution. (**A**) A Heat-map of cellular populations in Coho fin was constructed based on transcriptomic signals from each fin sample (individual rows represent individual fin samples) with yellow indicating higher proportions and purple lower proportions of particular cell type signatures (generated in GSEA). (**B**) The correlation plot shows a significant negative association between *Lsal* reads (FPKM) and plasma cells, activated DCs, Treg T cells, M1 macrophages, CD4 + T cells. In contrast, *Lsal* reads were positively associated with MCs (resting and activated), CD8 + cells (CTLs), M2 macrophages, neutrophils and naïve B cells (FPKM).

The contribution and association of cellular effectors to the rejection response in Coho salmon has been previously discussed^12,23^; however, these descriptions relied on routine histopathology. Here we paired functional transcriptomic profiling and histopathology with deconvolution using GEDIT^30^ to infer cell-type compositions in *Lsal*-infected fin. This program relies on human reference data sets, limiting the analysis to a certain degree as salmon has specific cell populations (i.e. NCCs, sacciform cells). However, most immunological cell types could be easily identified in the Coho transcriptome using this approach including T cells, B cells, neutrophils, eosinophils, macrophages (Mθ; M0, M1, M2), mast cells (MCs; activated and resting), and dendritic cells (DCs; activated and resting) (Fig. 6). Furthermore, manual curation of the transcriptome revealed significant association of NCCs with *Lsal* in Coho fin (Table 1).

**Table 1:** Cellular effectors involved in rejecting *Lsal* in *Okis* fin based on significant differential expression profiling of cellular markers and known effector molecules.

Cellular effector	Marker	Time	Function
NCCs (Teleost NK-cell equivalent)	↑ *nccrp-1*	1 → 7 dpi	Antigen-dependent cytotoxicity
	↑ *graa*	4 → 7 dpi	Activates cell death
	↑ *aep1*	1 → 7 dpi	Pore-forming protein
T cells	↑ *cd4 *	7 dpi	Activation of Th cells
	↑ *cd5 *	7 → 10 dpi	Th17 regulation
	↑ *cd33 *	7 dpi	T cell inhibition
	↑ *cd59*	1 dpi	T cell activation
	↓ * cd82*	7 → 16 dpi	Co-stimulatory with TCR/CD3
	↑ *cd166*	7 dpi	T cell activation
	↑ *cd226*	7 dpi	CTL/NK signalling
	↑ *cd247 *	10 dpi	TCR/CD3 complex
	↑ *cd276 *	1 dpi	Enhances CTL
	↑ *cxcl11 *	1 → 10 dpi	Chemotactic for activated T cells
B cells	↑ *cd22*	1 → 10 dpi	Lymphocyte interactions (B and T cells); regulator of B
	↓ *cd22*	16 dpi	cells
	↑ *cd53*	1 → 10 dpi	Mature B cells
Neutrophils	↑ * cxcr1*	4 → 10 dpi	Receptor for IL8, powerful neutrophil chemotactic factor
	↑ * ileu*	1 → 10 dpi	Regulates activity of neutrophil proteases
	↑ * lect2*	1 → 10 dpi	Neutrophil chemotaxis
	↑ * ncf1*	1 → 10 dpi	Necessary for superoxide production
	↑ * ncf2*	1 → 10 dpi	Necessary for superoxide production
Mast cells	↑ *fcεriγ*	1 → 16 dpi	Transducing subunit of FCεRI
	↑ *cd63*	1 → 10 dpi	MC degranulation
	↑ *ckit*	1 → 7 dpi	Tyrosine kinase growth factor receptor; role in MC development; enhances degranulation
	↑ *fyn*	4 → 10 dpi	Regulates MC degranulation
	↑ *lyn*	1 → 16 dpi	Interacts with FCeRI
	↑ *syk*	6 → 10 dpi	Protein kinase, transducer of FCeRI signalling
Dendritic cells	↑ *cd209*	1 → 10 dpi	Presents Ag to T cells via MHII
	↑ *c1qb*	7 dpi → 16 dpi	Bonafide DC marker; regulates hyperactive allergic responses
Macrophages	↑ * arg1 *	4 → 10 dpi	Alternatively activated macrophage marker
	↑ *mrc1*	4 → 10 dpi	Endocytosis of glycoproteins; cognate chitin receptor

Cell populations associated with the Coho response to *Lsal* were Mθ, MCs, neutrophils, B cells, CD4^+^ and CD8^+^ T cells (CTLs), DCs, and eosinophils, and there were interesting patterns in the populations throughout the infection. For example, activated MCs, naïve B cells, M2 Mθ, neutrophils, and eosinophils were strongly represented from 1 to 10 dpi (Fig. 6A), and as the infection proceeded, there was a shift from activation to resting MCs and DCs. Naïve CD4^+^ and T_reg_ cells, M1 Mθ, and plasma cells were a feature of control fin and at 1 and 16 dpi. To evaluate the association of these different cell types with presence of the parasite, we compared the number of *Lsal* read counts with the proportion of cellular effectors. A negative association was detected between *Lsal* read counts and plasma cells, T_reg_ cells, M1 Mθ, and activated DCs (Fig. 6B).

In contrast, there was a significant positive correlation with activated MCs, naïve B cells, CTLs, M2 Mθ, eosinophils, and neutrophils, indicating these cells were directly related with the presence and response to salmon lice (Fig. 6B). Notably, our data indicate a M2-dominant Mθ signal in *Okis* fin as early as 4 dpi. Known to accumulate in response to parasites in mammals^31^, M2 cells are known as the “default” mode of tissue-resident Mθ and are primarily maintained by TGF-b. Moreover, there was also a significant difference in the number of activated and resting MCs in controls at every timepoint except 7 dpi (Fig. 5B), corroborating enrichment of mast cell immunity as determined by GSEA.

**Figure 7:**
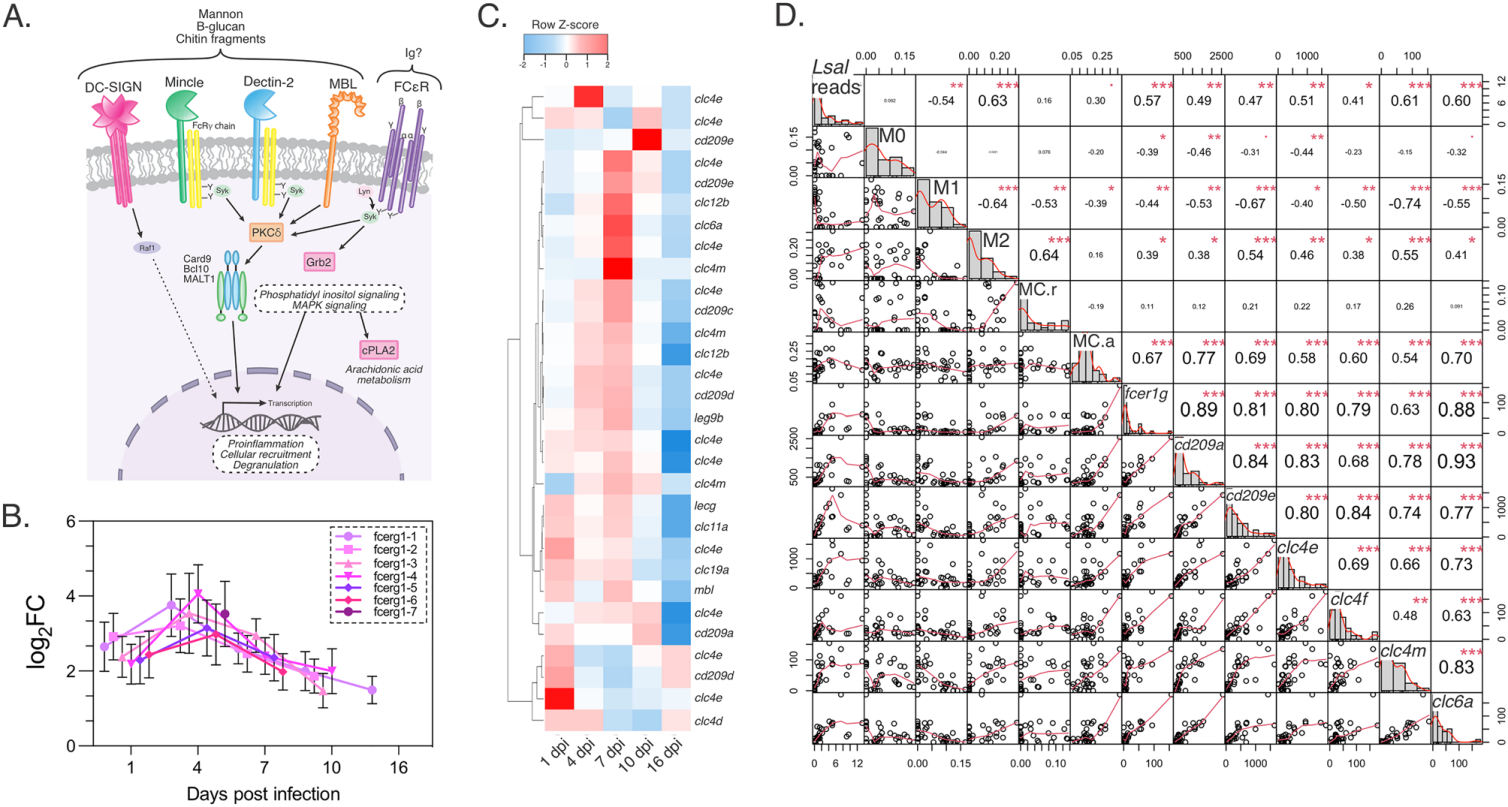
Markers of sugar/chitin moiety recognition were significantly induced in *Okis* fin. (**A**) Several classes of Pattern recognition receptors (PRRs) were induced in the fin transcriptome, including the C-type lectin receptors (CLRs) DC-SIGN, Mincle, Dectin-2, and MBL. (**B**) We also observed the induction of several isoforms of the IgE receptor subunit, *fcerg1*. (**C**) Expression profiles of CLRs demonstrate amplification of the response until 7 dpi. (**D**) Correlational analysis revealed a significant positive association between *Lsal* reads, *fcer1g*, M2 M0s, activated MCs, and the various classes of CLRs.

The dominant mechanism of MC activation in mammals is through cross-linking of the IgE receptor FceRI, which initiates signal cascades leading to a biphasic response of immediate degranulation followed by production and further release of prostaglandins, leukotrienes, cytokines, chemokines and growth factors^32^. Significant and sustained overexpression of several isoforms of the FceRI gamma subunit *fcerg1* observed in *Lsal*-infected fin (Fig. 7A), together with other profiles of MC regulation such as *cd63*, *cd300lf*, and *c-kit*^32^, support MC-driven hypersensitivity as a dominant driver of parasite rejection (Fig. 5). A role for MC-driven release of pre-formed mediators (e.g., proteases, prostanoids, leukotrienes, heparin, cytokines, chemokines, C3a, C5a, and growth factors) together with enhanced neutrophil infiltration in tissue graft rejection has been described in the skin of mammals^33^. Indeed, a feature of initial responses to *Lsal* in addition to a cytokine storm was significant infiltration of neutrophils concomitant with enrichment in arachidonic acid metabolism, complement and coagulation signaling, leukotriene signaling, and epidermal growth factor signaling (Figs. 2, 4). Furthermore, simultaneous overexpression of several chemotactic factors specific to neutrophils including *cxcl8*, *ncf1/2* and *lect2,* confirmed these cells as a key cellular effector against *Lsal* as suggested previously^12,23^. Throughout the progression of wound healing, the initial inflammatory phase depends on timely neutrophil recruitment and the associated regulators to prevent uncontrolled neutrophil activation^34^. Chemoattractants released by activated platelets in the wound microenvironment are critical for neutrophil recruitment and involve producing several growth factors and chemokines. Overexpression of several of these molecules including *vegfc*, *vegfa*, and *tgfb1* at 1 dpi in *Lsal*-infected fin supports a similar inflammatory phase important in wound healing cascades that intensified until 7 dpi.

Pioneering work on *Salmoniformes* demonstrated eosinophilic MC-like cells in the skin that were later shown to possess analogous functions to mammalian MCs, including recruitment to the site of injury, degranulation of inflammatory mediators and chemotactic substances in response to noxious stimuli, and in particular ectoparasites^35,36^.

**Figure 8:**
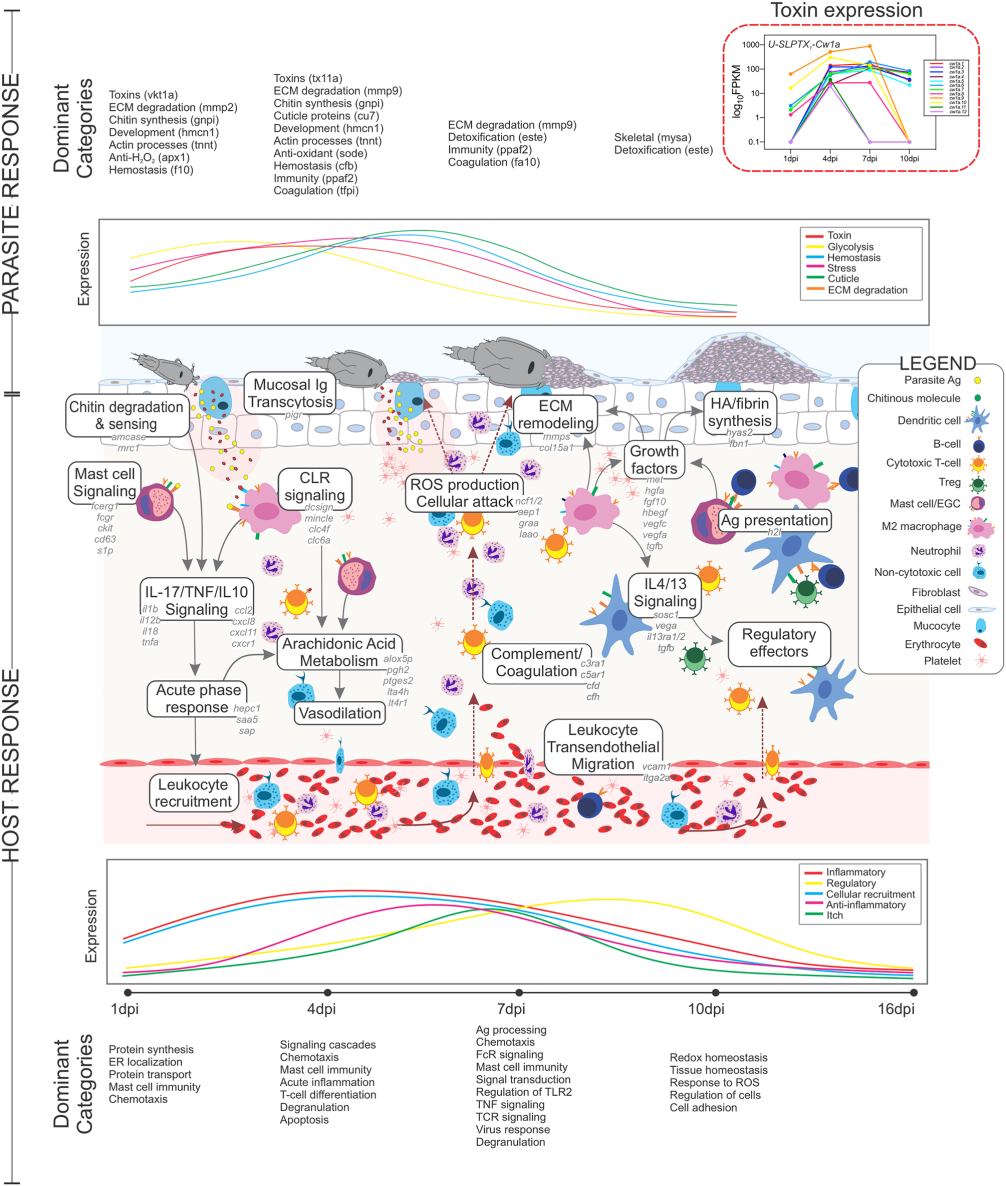
Schematic representation of the host-parasite interaction between *Okis* and *Lsal* through the period of parasite rejection (1 16 dpi). Based solely on the data reported herein.

More recent work described a functional MC equivalent in another teleosts species (*Danio rerio*, zebrafish)^37^ that possesses an analogous high-affinity immunoglobulin epsilon-like receptor (FCeRI), which contributes to allergic responses following TLR/MyD88-mediated signalling pathways in vivo^38^. Teleosts do not produce IgE making the existence of the associated receptor somewhat enigmatic^39^. However, IgG stimulation of the FCeRI receptor has been demonstrated in mammals, and Da’as et al. demonstrated cross-reactivity between zebrafish and human FCeRI receptors and IgE^38^. Thus, we hypothesize that allergic inflammation driven by FCeRI-dependent MC-degranulation potentiates and amplifies the aggressive recruitment of effector cells that act to both attack and neutralize the parasite while also supporting epithelial hyperplasia that results in encapsulation and eventual rejection by Coho salmon. Human and murine MCs are known to express C3a and C5a receptors^40^, serving to amplify allergic responses. Significant upregulation of both *c3ar1* and *c5ar1* in Coho fin concomitant with evidence for MC involvement suggest a similar mechanism whereby initial immunoglobulin-mediated degranulation of MCs amplifies the response through a positive feedback loop via the generation of C3a and C5a and subsequent activation of their GPCRs (Fig. 8).

In addition to the expression of *fcerg1*, the fin response was also dominated by the expression of lectin receptors (Fig. 7B), including *mbl*, *lecg*, and c-type lectin receptors (CLRs), and this expression was sustained through to 10 dpi, indicating they were associated with louse recognition/rejection. Specifically, there were several classes of CLRs in the Coho response (Fig. 7B,C): some that are known to act through indirect signalling (e.g., CLEC4E, CLC6A) by associating with other ITAM-containing adaptor molecules such as FcRg, as well as those bearing intracellular ITAM-like motifs (e.g., CD209, DC-SIGN)^41^. In the current work, there was an overabundance of *clec4e* isoforms in infected Coho fin (Fig. 7C). CLEC4E, known as macrophage-inducible C-type lectin (Mincle) is an indirect CLR known to bind to fungi and drive innate and adaptive responses, including the development of Th1 and Th17 responses^42^. Generally, CLRs are known to be essential PRRs for recognizing various mannose- and fructose-containing glycans present on the surface or excretory/secretory products of different parasites^43^, and CLRs are downregulated in response to salmon lice in susceptible species^44^.

Several glycan moieties have been identified in parasites, including protozoa, nematodes, trematodes and cestodes, which are likely targets for CLRs^43^. Comparatively, there is little known about what *Lsal* antigens might be interacting with host CLRs. Notwithstanding, significant positive association of CLRs including *clec4e/mincle* with *Lsal* in Coho fin (Fig. 7D), concurrent with upregulation of other important mediators of CLR signaling such as *fcr*g, *irf5*, *syk*, *card9,* and *bcl10,* supports this as a second dominant innate sensing mechanism for *Lsal*. Furthermore, a significant positive association between the different CLRs and the presence of M2 Mθ and activated MCs (Figs. 6B and 7D) indicate the PRRs are expressed by these cells. Interestingly, we observed significant overexpression of alpha-*N*-acetylgalactosamine-specific lectin (*lecg*), a lectin known to bind to and negatively impact parasite virulence in *Trypanosoma brucei*^45^. Subsequent functional association studies are required to determine the cellular source and role of lectins during the rejection of *Lsal*. For example, the presence of sacciform cells in Coho fin (Fig. 2) presents a novel potential source of PRRs. Others have shown that fugu (*Takifugu rubripes*) sacciform cells produce and secrete kalliklectin, a mannose-binding lectin only found in teleosts^46^. The significant induction of several classes of lectins paired with the presence of sacciform cells in Coho fin may represent a unique anti-parasitic PRR mechanism that necessitates subsequent investigation.

### Th1-type parasite rejection cascades peak at 7 dpi

A dominant feature of Coho fin during rejection of salmon lice is the production of granulomas that encompass and fully envelop the attached parasite by 10–16 dpi. Our transcriptomic profiling suggests this response is initiated by a concerted anti-parasite response mediated by chitin- and lectin-binding PRRs associated with tissue-resident Mθ, and FCeRI-signaling which is driven by tissue-resident MCs (Fig. 8). Contrary to anti-parasitic responses in other models where Th2-type profiles involving IL4 signaling often dominate^47^, our data supports the importance of a Th1-type inflammatory response in potentiating salmon lice rejection, followed closely by the initiation of regulatory profiles (Figs. 5, 6, 7, 8).

Th1-type responses are typically associated with defense against intracellular pathogens^47^. Contrary to this notion, our study demonstrates that an intracellular, antiviral-type response is directed towards attached salmon lice in Coho salmon. This included the early activation of a cytotoxic lytic activity profile by effector cells such as NCCs and CTLs that were positively associated with the presence of *Lsal* (R = 0.44, *p* = 0.012; Fig. 6). Although teleost-specific effector cell signatures such as NCC cells were not recognized using GEDIT, manual curation of DETs showed early overexpression of *non-specific cytotoxic cell receptor 1* (*nccrp-1*) in Coho fin by 1 dpi, suggesting NCCs were also involved in the response. NCC cells are the evolutionary precursor to NK cells in higher vertebrates and kill many target cells, including virus-transformed cells and protozoan parasites^48^.

The presence of CTLs was positively correlated with profiles suggesting the presence of neutrophils (*p* = 0.029), resting MCs (*p* < 0.0001), and M2 Mθ (*p* < 0.0001). Thought to respond exclusively to intracellular pathogens, CTLs and NK cells execute cell-mediated cytotoxicity (CMC) either through antibody-dependent or independent pathways, and these pathways are conserved among vertebrates. In fish, cellular cytotoxicity has been associated with the resolution of infection with intestinal parasites in gilthead sea bream, and neutralizing protozoans in tilapia^48^.

A paradox of this study is the apparent involvement of MCs in parasite rejection in the absence of both IL-4/13 and IgE, hallmark molecules involved in Th2-type anti-parasitic signaling and allergic immune pathways^29^. Specifically, our transcriptomic data indicates Coho fin is in a Th1-biased state throughout the cellular response to attached salmon lice, in concert with MC activation and regulation. While IL4/13 signaling was enriched throughout the infection; there was an absence of *il4/13* expression and a lack of the cognate Th2-associated cytokine milieu (Fig. 8). Furthermore, the decoy receptor, IL-13Ra2, which acts as an IL-13 scavenger receptor and inhibits downstream Th2-type responses was induced by 1 dpi, supporting earlier work that demonstrated that this receptor was downregulated in susceptible salmonids during *Lsal* infection^15^.

### Chitin degradation and presentation pathways in Coho fin

To elicit a response, large inert chitin molecules must first be degraded. In mammals, this is achieved by acidic mammalian chitinases and chitotriosidases produced by macrophages and epithelial cells at the site of parasite infection^49^. These enzymes, in turn, interact with PRRs such as *mannose-binding macrophage receptor 1* (*mrc1*) to initiate pro-inflammatory mediator secretion, resulting in the production of chemokines to recruit cellular effectors to the site. MRC1 has been described as one of the three innate immune receptors that bind to chitin moieties and mediates various immune responses to chitin in mammals^50^.

Chitin exposure induces IL-12, TNF-a, and IL-18 production in mammalian models, resulting in enhanced IFN-g production by NK cells^51^, as well as enhanced T cell activity. A strong pro-inflammatory, phagocytosis-dependent response characterized by inflammasome activation and IL1b secretion is observed after exposure to moderate-sized chitin fragments (e.g., chitosan)^52^, and this is shown to be regulated by keratinocytes in mammals^53^. We report that a similar mechanism maybe occurring in Coho fin, whereby significant expression of *mrc1, chia*, *lys, il12b*, *tnfa*, *ccl2* and *il18*, together with signatures of M2 Mθ, NCCs and CTLs, potentiates the initial Th1-response driven by chitin sensing as described above.

Chitin is also a potent innate immune modulator with the ability to stimulate both Th1- and Th2-type responses. For example, chitin exposure inhibits the production of IL-4 and IL-10 by upregulating IFN-g production by NK cells in spleen culture models^51^, and chitin exposure adjuvates immunity to attenuated *Mycobacterium bovis*^54^. Furthermore, chitin exposure is known to reduce allergic Th2-type responses (i.e., production of IL-4) in murine models concomitant with elevated levels of IL-12, TNF-a and IFN-g. In contrast, other studies have shown that chitin induces Th2-type responses characterized by eosinophil and basophil responses and upregulation of IL-4. These conflicting data are thought to be a function of the allergen itself and demonstrate that the modulatory capabilities of chitin are complex and must be considered in light of the chitin preparation (size), route of administration, and dose^55^. Here we provide compelling evidence that initial Th1-type anti-*Lsal* responses in Coho salmon is driven in part by chitin signalling pathways. Susceptibility to *Lsal* may reflect an alternate response to chitin, as chitin can initiate multiple cascades. Interestingly, the main chitin degradative enzymes in vertebrates, acidic mammalian chitinase (*chia*) and lysozyme (*lys*), are downregulated in susceptible Atlantic salmon^56^, implying that chitin is not being processed in this susceptible species for effective inflammatory activation, as is the case in our resistant model.

As mentioned, several potential cells may respond to chitin in Coho fin, including epithelial cells or tissue-resident APCs. For example, epidermal cells are critical in sensing chitin in mammals, with chitin exposure inducing significant TLR-dependent secretion of IL6, IL8 and TSLP^53^. Although we did not detect expression of *tslp*, significant induction of *il8* and *il6ra/b,* as well as the TSLP receptor, *il7r*, was a dominant feature in *Lsal*-infected Coho fin. Interestingly, despite its critical role in chitin-sensing, we did not observe upregulation of TLR2. However, there was significant downregulation of *tlr2* at the height of the response at 7 dpi, indicating a regulatory effort by Coho to dampen the response and reduce potential immunopathology associated with persistent TLR2 signalling. There was also significant downregulation of *tlr3*, a PRR typically associated with sensing of dsRNA found in MC endosomes^57^. In teleosts, TLR3 is also stimulated by bacterial PAMPs such as b-glucans^57^, implying a potential role for TLR3 in the anti-louse response in Coho salmon.

Notably, endosomal TLR3 degradation is a hallmark of host immune modulation by *Fasciola hepatica*, facilitated by parasite-derived cysteine proteases captured by macrophages^58^. Indeed, we observed significant upregulation of *LsalCL1,* a major virulence factor of *Lsal*^7^*,* at 7 dpi (Fig. 8 and Supp Fig. 3). The concomitant downregulation of *tlr3* may reflect a negative compensatory response by the host to restrict parasite virulence. However, as evidenced by the significant pathology associated with unrestricted Th1-type inflammatory pathways, the resolution of immune responses is a critical component of host resistance. Thus, suppression of *tlr3* expression likely represents host-induced regulation of inflammation to limit immunopathology once parasite killing was initiated. Taken together, our data supports initial immune recognition (e.g., within 24 hpi) by resident MCs, sacciform cells, and Mθ in Coho fin. However, transcriptomic upregulation of markers for neutrophils, B cells, dendritic cells and T cells within 96 h suggests the sustained response is a coordinated effort between multiple cellular effectors (Fig. 6).

### Interplay between parasite and host responses

Host-parasite interactions result from both parasite and host responses, and thus either interacting partner cannot be considered mutually exclusive. Using a dual RNA sequencing approach has been valuable in this context by generating information on pathogen virulence pathways, host interacting pathways, and informing as to novel interventions. Here we provide the first interaction data on the response of *Lsal* during infection and rejection by a resistant host. The response of *Lsal* at 1 dpi was enriched by genes involved in processes such as glycolysis, tissue development, coagulation, and detoxification (Fig. 8 and Table 2). At 4 dpi, and concomitant with frontal filament deposition and granuloma formation, cuticular development was significantly increased (e.g., *cu07, cu146, cu12, cu168, resil*) genes (Supp Fig. 3).

**Table 2:** Manually curated categories of DETs in *Lsal* at 1 10 dpi, including the putative biological function and representative transcripts.

Timepoint	Functional category	Transcript	log2FC
1 dpi	Glycolysis	*gnpi*	6.24
	Muscle development	*mhc*	3.45
	Coagulation	*tfpi*	12.31
	ECM degradation	*mmp9*	10.28
	Toxins	*cal9.1a*	12.39
	Detoxification	*pxdn*	8.78
	Actin binding	*hmcn1*	10.65
4 dpi	Cuticular development	*cu15*	8.28
	Chitin binding	*obste*	6.11
	Coagulation	*cfb*	5.27
	ECM degradation	*mmp9*	7.68
	Toxins	*cw1a*	9.16
	Actin binding	*acl6a*	4.95
	Virulence	*pcema1*	7.06
	Complement inhibitor	*prop*	6.58
	Immune response	*ppaf2*	7.52
7 dpi	Toxins	*cw1a*	9.19
	ECM degradation	*lce*	7.53
	Cuticular development	*cu07*	7.19
	Coagulation	*cfb*	7.04
	Detoxification	*txndc17*	5.8
	Proteases	*catl*	2.61
10 dpi	Amino acid degradation	*hgo*	1.33
	Microtubule process	*dynlrb2*	1.24

Expression is represented as log_2_fold-change.

Furthermore, at 4 dpi the response of *Lsal* also showed an upregulation of several detoxification enzymes, including glutathione peroxidases (e.g., *gpx4*) and superoxide dismutase. The reactive oxygen species (ROS) hydrogen peroxide (H_2_O_2_) is highly efficient at inducing cell death and has been demonstrated to be strongly destructive against intracellular parasites such as trypanosomes^59^. The pathway of H_2_O_2_ production is catalyzed by the enzyme L-amino acid oxidase (L-AAO), which mediates the oxidative deamination of L-amino acids to produce a-keto acid concomitant with H_2_O_2_, and ammonia. Simultaneous with a response to neutralize ROS, the Coho fin transcriptome was characterized by induction of *laao* at 4 dpi (Supp Fig. 3). By 10 dpi, we observed peak induction of many proteases (e.g., trypsins, cathepsins) that have been described in the secretome of *Lsal* to be putative virulence factors^7^ (Supp Fig. 3). A majority of these proteases function in ECM degradation (e.g., *lce, mmp2, mmp9*), and are likely playing a role in digestion based on functional domain homology with orthologues in ticks and mites^60^.

Interestingly, we observed several classes of venom-like toxins in the *Lsal* transcriptome, including astacins (e.g., *nas-6, nas-7, nas-8, nas-15*), conotoxins (e.g., *cal9.1a*), scoloptoxins (e.g., *cw1a*), and venom allergens (e.g., *va5*) (Fig. 8 and Supp. Fig. 3). There were numerous examples of astacin-like genes overexpressed by *Lsal* in the present study, and these have previously been described in the secretome of adult salmon lice (reviewed in^7^). The M12 family of metalloendoproteases have been recruited into the venoms of many genera including spiders and ticks^60^, and are thought to aid in the spread of other virulence factors by degradation of the ECM^61^. Moreover, the transcriptome of *Lsal* was populated by 12 different isoforms of the scoloptoxin *U-SLPTX*_*1*_*-Cw1a* which increased in expression until 7 (Fig. 8).

Scoloptoxins are < 10 kDa, cysteine-rich peptides that have undergone extensive functional radiation across several lineages^61^, but have not been described in ectoparasitic copepods previously to our knowledge. The function of toxins in *Lsal* requires further characterization; however, based on significant conservation among these proteins within Arthropoda, they likely share functional similarities with their homologs, including putative roles in anticoagulation, tissue degradation, and neurotoxicity^62,63^. Interestingly, venoms are known to activate mast cells, and mammalian MCs can enhance host resistance to the toxicity of several venoms, supporting the notion that allergic inflammation can enhance innate and acquired host resistance to ectoparasites such as ticks^60,64^. Thus, it is plausible that *Lsal* toxins are part of what drives initial MC recognition and the ensuing aggressive inflammatory cascades leading to parasite rejection, and that MC activation results in rapid release of pre-formed molecules that can act to both potentiate inflammatory cascades (e.g., prostaglandins), while also degrading and neutralizing the effects of toxins (e.g., heparin, proteases) in an evolutionarily conserved mechanism for combating toxicity of pathogens.

## Conclusion

This is the first transcriptomic profiling of resistant Coho salmon during infection and subsequent rejection of the salmon louse. Our approach generated a high-resolution dataset that enabled a comprehensive molecular characterization of the rapid rejection phenotype observed in Coho salmon. This response was present irrespective of size or time in saltwater. Specifically, we have produced evidence for chitin and/or carbohydrate PAMP sensing likely by tissue-resident MCs and Mθ that initiate and potentiate the aggressive inflammatory cascade resembling allergic inflammation, resulting in engulfment and rejection of the attached parasite.

The salmon louse antigen responsible for driving initial host recognition has not yet been identified. Our data demonstrates that *Okis* recognizes the attached larvae by 1 dpi. However, this is in the absence of the penetrating frontal filament, which takes about 72 h to form once the copepodite has successfully found a host^65^. Upon locating their host, *Lsal* copepodites first utilize modified secondary antennae as grapples to anchor before frontal filament extrusion^65^. Furthermore, we have identified significant production of toxins by *Lsal* larvae as early as 1 dpi, homologues of which are known to be highly immunogenic in other vertebrate hosts^60,64^. Thus, the aggressive anti-louse response observed in Coho salmon is likely a multipronged response to parasite-derived carbohydrates, chitinous-like molecules, and/or toxins. Understanding the divergence in these fundamental mechanisms that enable Coho salmon while conversely disables Atlantic salmon to respond to *Lsal* will follow this work and illuminate targets for development of novel control strategies to combat the global impacts of salmon lice.

## Materials and methods

### Ethics statement

All fish handling and procedures were approved by the Department of Fisheries and Oceans Canada (DFO) Maritimes & Gulf/CFIA Regional Animal Care Committee (AUP 15–26 and 16–10) and carried out under the direct supervision of a trained DFO Canada scientist in strict compliance with regulations set out by the Canadian Council for Animal Care (http://www.ccac.ca/). All experiments were performed in accordance with relevant guidelines and regulations, and followed recommendations described by the ARRIVE guidelines for conducting research on animals.

### Experimental procedures

Coho salmon *Oncorhynchus kisutch* (*Okis*) were transferred at ~ 1 g from Nanaimo River Hatchery, Nanaimo, British Columbia, to a Level III Quarantine Facility in Charlottetown, Prince Edward Island, Canada. Fish were acclimated to ambient recirculating conditions (freshwater, 10–12 °C) over several hours in a 400 m^3^ tank. When the population average weight reached 10 g, along with visual indicators of smoltification such as loss of parr marks, Instant Ocean was slowly added to the partially recirculating system such that salinity was increased by 4–5 ppt per day to achieve 32–33 ppt in 7 days. Fish were fed to satiation 2–3 times daily with a commercially available diet (Skretting; Skretting, #2 Crum initially and later 2.3 Nutra RC).

Water conditions were maintained at 12 ± 0.1 °C with a salinity of 32–33 gL^-1^ and conditions were monitored daily. After 4–5 months in freshwater and immediately following transition to full salinity, Coho (n = 40–48) were arbitrarily transferred to 4 identical 28–30 L tanks to conduct the parasite challenge using duplicate tanks for infection and control treatments.

### Lsal challenge experiments

Nauplii I of *Lepeophtheirus salmonis salmonis* (*Lsal*) were obtained from Huntsman Marine Science Centre, St. Andrews, New Brunswick, and incubated in aerated full strength ocean water at 10 °C. Once evidence of molting to copepodite was observed, cultures were enumerated and diluted such that ~ 60–80 copepodites per fish could be delivered during the experimental exposure challenge.

Two challenge experiments were designed to test the hypotheses that resistance to *Lsal* in Coho salmon is related to: (a) host size, and (b) time in saltwater (TIS). The first challenge involved Coho post-smolt that were acclimated to 33 ppt for 24 h (CS1: weight 11.7 ± 3.6 g, length 10.4 ± 1.3 cm), while the second involved Coho post-smolt (CS2: weight 18.6 ± 4.9 g, length 12.1 ± 0.9 cm) that had been exposed to 33 ppt salt for 30 days. In both challenges, fish were exposed to infective *Lsal* copepodites at a concentration of 60–80 fish^−1^ for 2 h (Fig. 1A). Water flow to the experimental exposure tanks was stopped just prior to adding lice, and fish were observed closely during the exposure time. Supplemental oxygen was available if saturation decreased below 95%. Jumping, flashing and other behaviors indicative of parasite attachment were observed. At each sampling point (Fig. 1A), fish were opportunistically removed by carefully dip-netting eight (8) animals from each of two replicate tanks and immediately transferred to individual vessels dosed with 200 mg/L tricaine methanosulfonate (Syndel, TMS-222). Information including weight (g), length (cm), and total lice abundance was captured. Infected fins were carefully dissected with sterile scissors, and any areas where attached lice were on the skin were biopsied using 5 mm biopsy punches (AcuPunch), keeping the louse attachment site in the center of the punch. Gills, spleen, anterior kidney, and liver were also sampled. All tissues were immediately placed in RNA*later* (Ambion) for RNA analysis, while duplicate tissue samples were immediately preserved in 10% neutral buffered formalin (NBF) for subsequent histological analysis.

### Histopathology

Necropsies (including macroscopic external and internal examinations) and tissue collections were conducted at the Level III Quarantine facilities while histologic slide preparation and slide evaluation were conducted at the Atlantic Veterinary College (AVC), University of Prince Edward Island, Charlottetown, Prince Edward Island, Canada. Samples of fin, gill, and skin were fixed in 10% neutral buffered formalin for a minimum of 48 h. Following fixation, specimens were decalcified using Cal-Ex Fixative/Decalcifier (Fisher Chemical) for periods ranging from 24 to 48 h. Specimens were then gross-trimmed for placement into standard tissue cassettes, following which they were processed for paraffin embedding using a Sakura Tissue-Tek VIP-6-A1 processor. Each resulting paraffin block was sectioned on a microtome. Ultra-thin sections (5 mm) were rehydrated in graded ethanol and distilled water and subjected to toluidine blue, and haematoxylin and eosin (H&E) staining. Stained sections were visualized using via brightfield microscopy on a Leica DM2500 brightfield microscope, with images captured using a PixeLINK PL-B686CU digital camera.

### RNA extraction, DNase treatment, evaluation of RNA quality and qRT-PCR

Total RNA was isolated from tissues using a modified chloroform-phenol extraction^8^. Briefly, fin samples were transferred to 1 mL of freshly prepared TRI reagent and homogenized with 2.4 mm ceramic beads for 30 min at 50 Hz (TissueLyser, Qiagen). Samples were then incubated at 55 °C for 30 min with 10 ml of proteinase K, followed by RNA isolation using RNeasy columns (Qiagen) without the optional column DNase treatment. Total RNA was eluted in 20–30 ml ultrapure water. Ten mg of total RNA was treated with *Turbo*®-DNase (Ambion) following instructions for routine treatment. The resultant purified, DNase-treated RNA was quantified by spectroscopy (NanoDrop 2000) and 1 ml of each sample was visualized using gel electrophoresis to check for degradation. Only samples with distinct 18S and 28S ribosomal bands were analyzed further by digital PCR analysis (Experion, BioRad).

The same samples used for RNA sequencing analysis were used for RT-qPCR validation targeting transcripts differentially expressed at various fold changes between treatments. Synthesis of cDNA was completed for 2 μg of total RNA in a 20 μl reaction using the iScript Reverse Transcription Supermix for RT-qPCR kit (Bio-Rad). Reverse transcriptase-free reactions were completed in parallel to confirm the absence of genomic nucleic acid contamination. Standard curves were generated for each target transcript (6-point, threefold dilution series) to confirm proper primer efficiencies. RT-qPCR amplification was performed using SsoAdvanced SYBR Green Supermix (Bio-Rad) in 11 μl reactions with 1 μl template input. The following thermal cycling regime was utilized for amplification: 95 °C for 30 s followed by 40 cycles of denaturation at 95 °C for 15 s and a combined annealing and extension step at specific primer pair Tm—5 °C (52–58 °C) for 30 s. A dissociation curve analysis was performed immediately post-amplification by increasing the temperature by 5 °C increments every 5 s starting from 65 °C until 95 °C to corroborate single product amplification previously determined by gel electrophoresis. All RT-qPCR reactions were completed using the same CFX384 Real-Time PCR Detection System (Bio-Rad). Reference gene stability and mean normalization of RT-qPCR data was completed on a log_2_ scale relative to reference genes elongation factor 1 alpha (*ef1a*) and ribosomal protein L13 (*rpl13*) using Bio*-*Rad's *CFX* Maestro software.

### Read processing, mapping, and differential expression analysis

Thirty-five RNA samples from the second experiment CS2 with RIN > 7 were used for sequencing library construction with the Illumina TruSeq stranded kit at Genome Quebec’s Centre d’Expertise et de Services (CES) in Montreal. Libraries were sequenced in two lanes of one flowcell using the HiSeq2500 instrument at CES. Samples were barcoded, and ~ 17 individuals were sequenced per lane. A total of 651,763,086 read pairs were obtained, with an average coverage of 20,190,113 read pairs per sample (~ 5 Gb per sample; S1 Table). Adaptors and low-quality bases were trimmed using Trimmomatic v0.36 (ILLUMINACLIP:2:30:10 SLIDING WINDOW:4:5 LEADING: 5 TRAILING: 5 MINLENGTH: 25).

Trimmed reads were mapped to the *O. kisutch* genome v2 (GCF 002,021,735) and the sea lice draft assembly (ASM18125v2) using Hisat v2.1.0^66^ with the –fr and –dta flags. StringTie v2.05^66^ was used to assemble individual transcriptomes (using the annotation provided by NCBI), and resulting GTFs were used to generate a merged transcriptome. Count data was generated using the prepDE.py script included with StringTie and were analyzed using DESeq2 and edgeR to identify differentially expressed transcripts (DETs) with an adjusted *p*-value of < 0.01 between infected (1, 4, 7, 10, 16 dpi) and unexposed control (1 dpi used as a common reference) fin samples. As the DESeq2 DET lists were > 98% fully encompassed with the EdgeR DET lists, the DESeq2 lists were used as the default DETs for the remainder of the analysis. Generated lists of DETs were compared using Venny v2.1 to create Venn diagrams to illustrate shared transcripts/GO terms across groups/time^67^.

To maximize the accuracy of the functional annotation of resulting DETs, the Uniprot and SwissProt databases were used to re-annotate all *Okis* and *Lsal* transcripts. Briefly, sequences for transcripts were generated using gffread v0.12.2^68^ with the merged transcriptomes as the input. The 2020_1 version of the UniProtKB/SwissProt was downloaded and sequences were blasted locally using blastx as implemented in the blast + v2.90 suite using an e-value threshold of 10^–10^. Results were filtered with in-house scripts to extract the blast-hit with the lowest e-value for every transcript, and SwissProt accession numbers were used to retrieve GO annotations (S4 File).

### Pathway and term enrichment analysis

Functional annotation and functional enrichment of DETs (FDR > 5%) was performed using a combinatorial approach with results obtained from the Database for Annotation, Visualization and Integrated Discovery (DAVID) framework^69^, as well as with g:Profiler^70^ with a fishers’ exact test and *p*-value of < 0.05 as significantly enriched. This provided a comprehensive list of enriched GO terms, KEGG pathways, REACTOME pathways and regulatory elements (transcription factors and miRNAs) involved in the response to *Lsal* over time.g:GOSt analysis in g:Profiler was performed on lists of *Okis* DETs from each timepoint ranked by fold-change^71^. Data sources included GO (Biological Process), KEGG, and Reactome. Lists of enriched GO terms were trimmed using Revigo to reduce redundancy^72^. The top 25 GO significant terms and enriched pathways (KEGG and REACTOME) were visualized using GOplot and ggplot2 in RStudio v3.6.2.

### Gene set enrichment analysis (GSEA)

Gene set enrichment analysis was performed using GSEA desktop application version 3.0 (Broad Institute), employing predefined gene sets from the Molecular Signatures Database v7.2 (http://www.gsea-msigdb.org/gsea/msigdb/index.jsp).

In the present study, GSEA was performed on a non-ranked list of normalized filtered differentially expressed transcripts (FPKM; > 10 read counts per transcript) obtained from the RNA sequencing described above using the collected gene-sets of KEGG, Reactome, and gene ontology (mm_GO) with the following parameters: 1000 gene set permutations, weighted enrichment statistics, gene set size between 15 and 500, and signal-to-noise metrics for ranking genes. Regulated gene-sets were considered statistically significant if false discovery rate (FDR) ≤ 20% (General GSEA for consideration of large numbers of sets) and nominal *p*-value ≤ 0.001. The GSEA-derived normalized enrichment score (NES) was used to determine the magnitude of up- or down-regulation of enriched gene-sets. Enrichment mapping was performed on GSEA results in order to visualize commonly enriched genes within the gene sets^71^. For each database of gene sets, GSEA was performed across all uniquely expressed transcripts in *Lsal*-infected *Okis*-fins compared to non-infected *Okis*-fins using the gene-set permutation.

### Functional enrichment visualization and annotation

Results from the GSEA and g:Profiler analysis were visualized using EnrichmentMap (v3.3.1) plugin in Cytoscape (v3.7.2, https://cytoscape.org/) and mapped as a network of gene-sets (nodes) where the nodes represent statistically significant terms and the links (edges) represent the degree of gene-set similarity. Combined Jaccard (50%) and Overlap (50%) metrics with the default cutoff of 0.375 were applied. The enriched gene-sets annotation were grouped by AutoAnnotate (v1.3.2) according to the Markov cluster (MCL) algorithm based on the edge weights of similarity coefficients, and were automatically annotated using the Wordcloud algorithm (v3.1.3) with the maximum of four words per label.

### Statistics

Where appropriate, results are expressed as the mean ± SEM (standard error of the mean). Statistical significance was determined using parametric two-way analysis of variance, followed by post hoc Tukey’s HSD.

## Supplementary Information

Supplementary Information 1.

Supplementary Information 2.

Supplementary Information 3.

Supplementary Information 4.

Supplementary Information 5.

Supplementary Information 6.

Supplementary Information 7.

Supplementary Information 8.

Supplementary Information 9.

## Data Availability

The RNA-seq datasets generated here that support the findings of this study are openly available in the SRA database of NCBI stated in the results/discussion (BioProject ID PRJNA765642). Further data or graphic visualization methodologies can be accessed through contacting the corresponding author (MDF).
