# Supplementary figures and images for: Rejection of Lepeophtheirus salmonis driven in part by chitin sensing is not impacted by seawater acclimitization in Coho salmon (Oncorhynchus kisutch)
Source: Sci Rep. 2023 Jun 15;13:9685. doi: 10.1038/s41598-023-36632-0 (PMC10272145; doi:10.1038/s41598-023-36632-0)

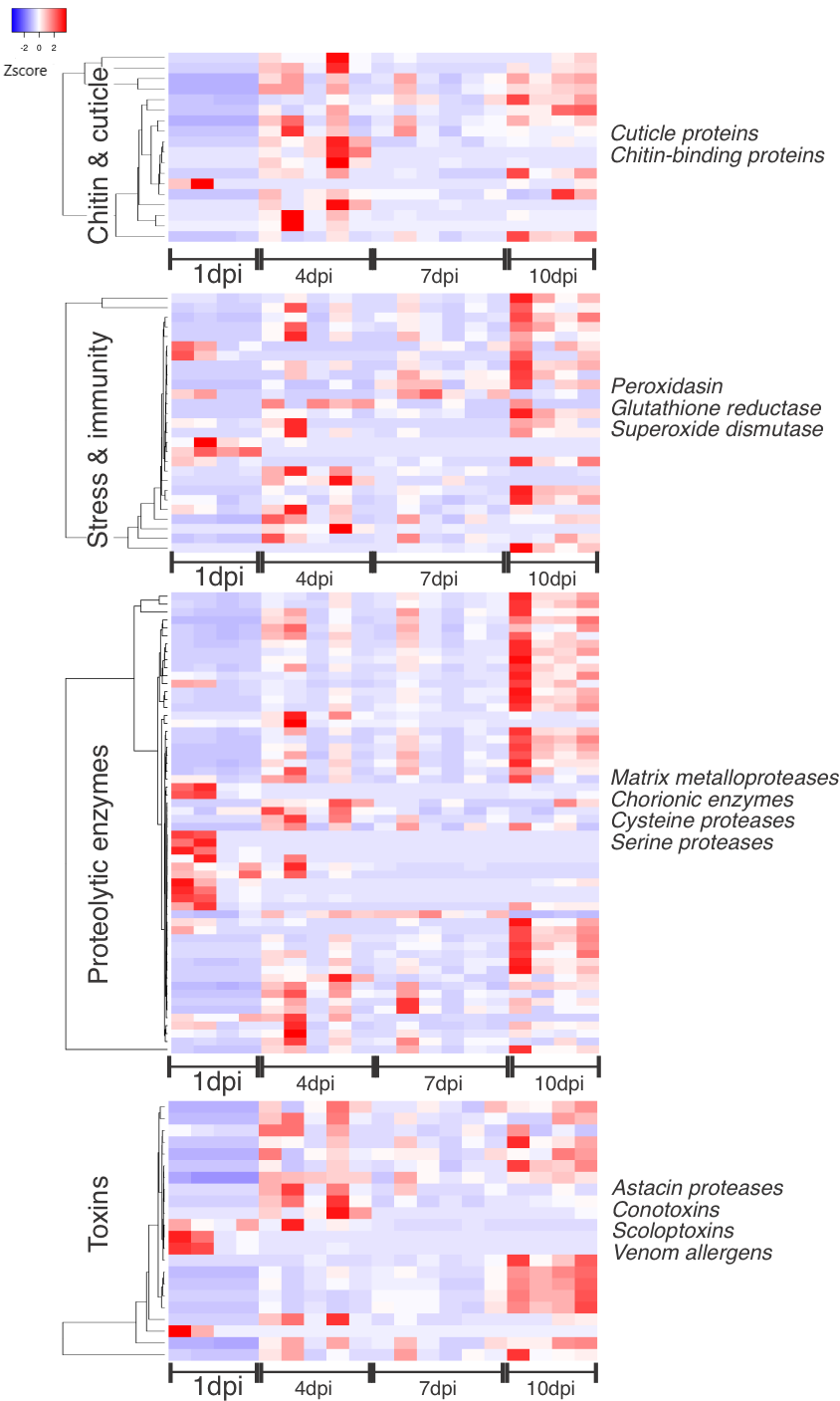

Supplement: Supplementary file 5 — Supplementary Information 5. [file 41598_2023_36632_MOESM5_ESM.tif]

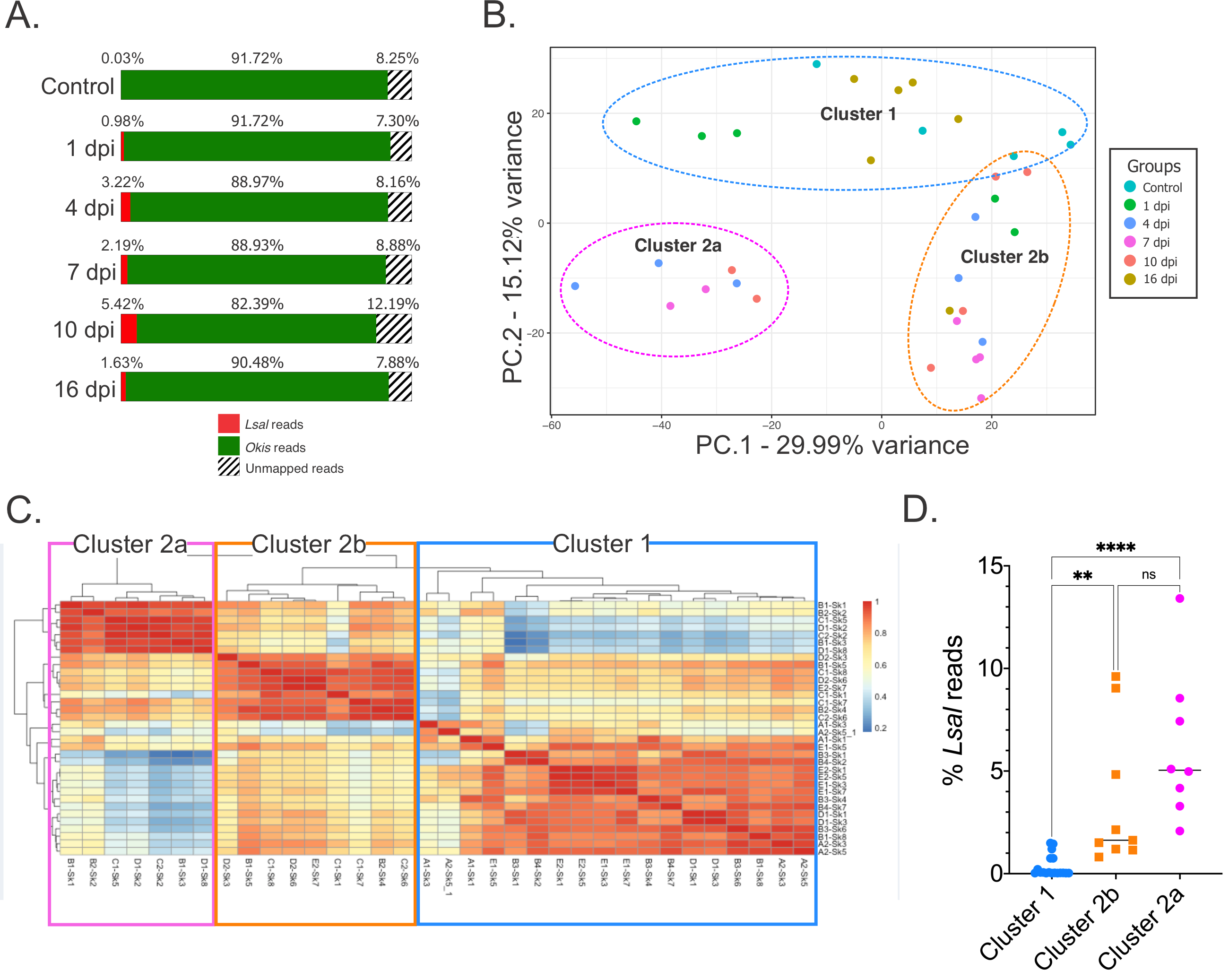

Supplement: Supplementary file 6 — Supplementary Information 6. [file 41598_2023_36632_MOESM6_ESM.tif]

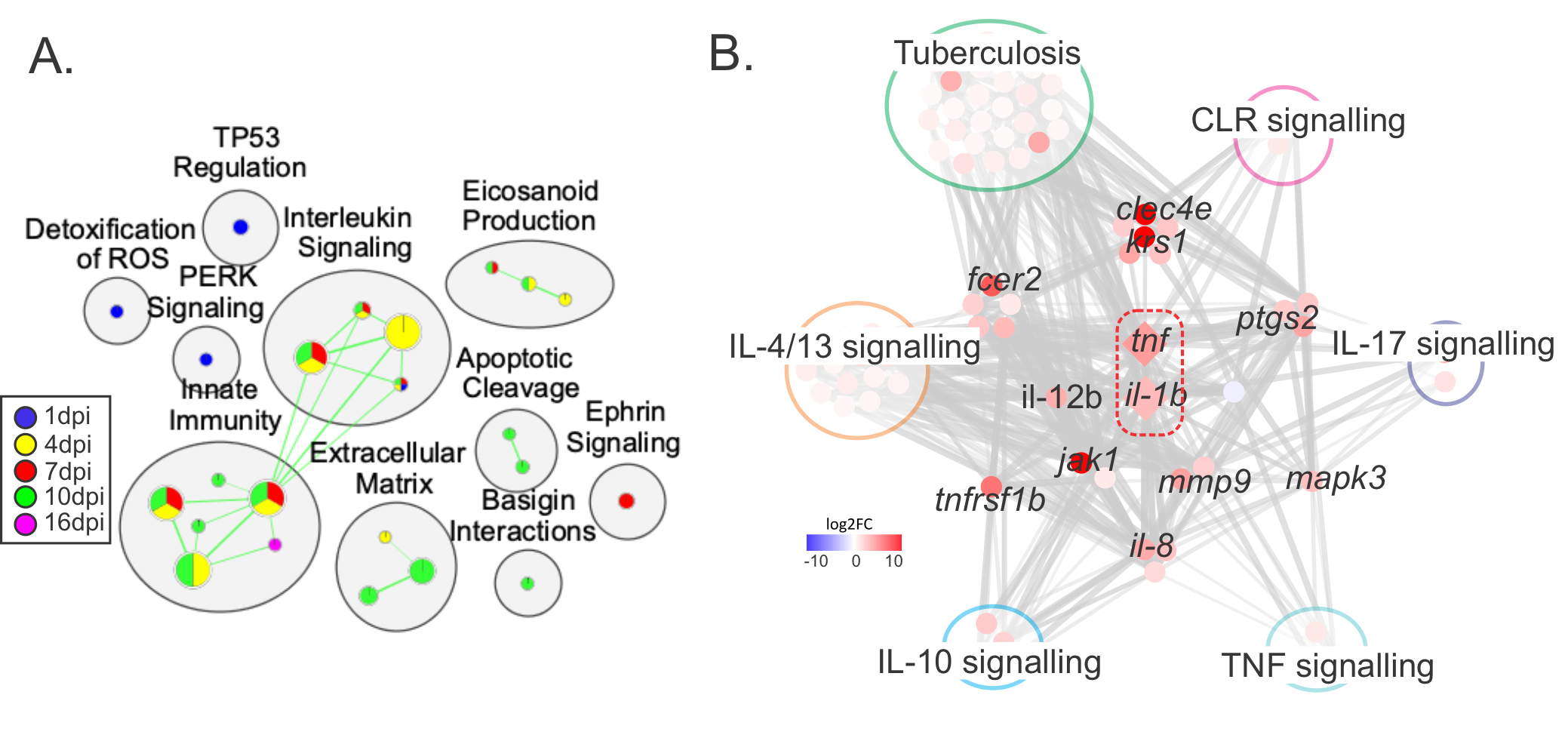

Supplement: Supplementary file 7 — Supplementary Information 7. [file 41598_2023_36632_MOESM7_ESM.tif]
