# Supplementary material for: Rejection of Lepeophtheirus salmonis driven in part by chitin sensing is not impacted by seawater acclimitization in Coho salmon (Oncorhynchus kisutch)
Source: Sci Rep. 2023 Jun 15;13:9685. doi: 10.1038/s41598-023-36632-0 (PMC10272145; doi:10.1038/s41598-023-36632-0)
Supplement: Supplementary file 8 — Supplementary Information 8. [file 41598_2023_36632_MOESM8_ESM.docx]

**Supplemental Figure 1: Transcriptomic profiling of Coho fin response to salmon lice.**

(A) Read counts (fragments per kilobase million) generated by RNA sequencing over time, showing the proportion of *Okis* (green) to *Lsal* (red) counts. Unmapped reads (black and white stripes) ranged from 7.30 to 12.19%. (B) Principal component analysis and distance clustering of FPKM showed that ~30% of the variation was explained by PC1 while PC2 explained ~15% of the variability in expression profiles. (C) Heatmap of each sample’s transcriptomic profile and its associated clustering. Sample identifiers were ‘Time[letter]’ (1 dpi, A; 4 dpi, B; 7 dpi, C; 10 dpi, D; 16 dpi, E), ‘tank’ [1,2 infected; 3,4 ctrl] and individual fish number [i.e. Sk4].(D) The number of *Lsal*-read counts (%) per sample was correlated with the samples found in each cluster to detect a significant contribution of parasite RNA (*p* < 0.001) on the divergence pattern of samples within cluster 2a and cluster 1.

**Supplementary Figure 2: Functional enrichment visualization and annotation in *Okis* fin during infection with *Lsal*.**

(A) EnrichmentMapper was used to visualize the most significantly enriched biological pathways (Reactome, KEGG) over time. Results from the GSEA and g:Profiler analysis were visualized using EnrichmentMap (v3.3.1) plugin in Cytoscape (v3.7.2, <https://cytoscape.org/>) and mapped as a network of gene‑sets (nodes) where the nodes represent statistically significant terms and the links (edges) represent the degree of gene-set similarity. The top 5 major pathways involved in the rejection response included interleukin signaling, innate immunity, extracellular matrix, eicosanoid production and apoptosis. Size of pie charts are affected by number of DEGs within a particular function, and colour is based on the specific time point these functions were enriched (B) As interleukin signalling was the most significantly enriched pathway in A, it was visualized as an interaction network showing expression as log2-transformed fold-change. As can be seen, the dominant hub genes (highest number of interacting points with other networks, and therefore central to the network) driving all the pathways were *tnfa* and *il1b*.

Supplementary Figure 3: **The *Lsal* response during active infection on Okis fin**.

The louse response was dominated by several key categories of genes, including chitin/cuticle, stress/immunity, proteolysis, and toxins. The general theme included activation of these genes immediately upon attachment at 1 dpi, followed by repression at 7 dpi. At 10 dpi there appeared to be significant induction of these genes, concomitant with granuloma formation by the host.
