# Supplementary material for: Rejection of Lepeophtheirus salmonis driven in part by chitin sensing is not impacted by seawater acclimitization in Coho salmon (Oncorhynchus kisutch)
Source: Sci Rep. 2023 Jun 15;13:9685. doi: 10.1038/s41598-023-36632-0 (PMC10272145; doi:10.1038/s41598-023-36632-0)
Supplement: Supplementary file 9 — Supplementary Information 9. [file 41598_2023_36632_MOESM9_ESM.docx]

## Supporting information

**S1 Table.** Raw reads obtained from RNA-sequencing.

| **Sample ID** | **Treatment** | **Number of reads** |
| --- | --- | --- |
| A1-Sk1 | Infected, 1 dpi | 20,016,505 |
| A1-Sk3 | Infected, 1 dpi | 25,414,755 |
| A2-Sk3 | Infected, 1 dpi | 21,151,771 |
| A2-Sk5 | Infected, 1 dpi | 14,914,661 |
| A2-Sk6 | Infected, 1 dpi | 22,870,197 |
| B1-Sk1 | Infected, 4 dpi | 21,639,960 |
| B1-Sk3 | Infected, 4 dpi | 18,039,813 |
| B1-Sk5 | Infected, 4 dpi | 16,463,460 |
| B1-Sk8 | Infected, 4 dpi | 20,068,466 |
| B2-Sk2 | Infected, 4 dpi | 20,566,176 |
| B2-Sk4 | Infected, 4 dpi | 17,407,145 |
| B3-Sk1 | Control, 4 dpi | 19,951,464 |
| B3-Sk4 | Control, 4 dpi | 20,226,127 |
| B3-Sk6 | Control, 4 dpi | 28,887,153 |
| B4-Sk2 | Control, 4 dpi | 21,687,926 |
| B4-Sk7 | Control, 4 dpi | 25,231,696 |
| C1-Sk1 | Infected, 7 dpi | 17,808,744 |
| C1-Sk5 | Infected, 7 dpi | 19,589,475 |
| C1-Sk7 | Infected, 7 dpi | 15,722,903 |
| C1-Sk8 | Infected, 7 dpi | 16,082,597 |
| C2-Sk2 | Infected, 7 dpi | 18,237,569 |
| C2-Sk6 | Infected, 7 dpi | 18,535,281 |
| D1-Sk1 | Infected, 10 dpi | 21,606,399 |
| D1-Sk2 | Infected, 10 dpi | 16,778,175 |
| D1-Sk3 | Infected, 10 dpi | 15,939,893 |
| D1-Sk8 | Infected, 10 dpi | 21,322,168 |
| D2-Sk3 | Infected, 10 dpi | 15,977,688 |
| D2-Sk6 | Infected, 10 dpi | 13,838,431 |
| E1-Sk3 | Infected, 16 dpi | 17,888,454 |
| E1-Sk5 | Infected, 16 dpi | 18,501,677 |
| E1-Sk7 | Infected, 16 dpi | 19,347,742 |
| E2-Sk1 | Infected, 16 dpi | 20,657,350 |
| E2-Sk5 | Infected, 16 dpi | 13,928,414 |
| E2-Sk7 | Infected, 16 dpi | 15,462,852 |
|  | Total | 651,763,086 |

**S2 Table. Results of gene-set enrichment analysis.**

| PATHWAYS | Enriched | | | | | Repressed | | | |
| --- | --- | --- | --- | --- | --- | --- | --- | --- | --- |
| Comparison | # gene sets | | FDR<25% | p<1% | p<5% | # gene sets | FDR<25% | p<1% | p<5% |
| 1 dpi vs. control | 530 | | 125 | 87 | 128 | 4100 | 129 | 302 | 745 |
| 4 dpi vs. control | 596 | | 102 | 80 | 125 | 4034 | 16 | 167 | 437 |
| 7 dpi vs. control | 654 | | 117 | 99 | 148 | 3976 | 45 | 83 | 238 |
| 10 dpi vs. control | 626 | | 135 | 121 | 159 | 4004 | 59 | 130 | 304 |
| 16 dpi vs. control | 178 | | 43 | 31 | 39 | 4452 | 5 | 106 | 319 |
| Time series (1🡪16 dpi) | 3860 | | 909 | 464 | 884 | 770 | 223 | 124 | 203 |
| REGULATORY | | Enriched | | | | Repressed | | | |
| Comparison | | # gene sets | FDR<25% | p<1% | p<5% | # gene sets | FDR<25% | p<1% | p<5% |
| 1 dpi vs. control | | 22 | 0 | 0 | 0 | 3151 | 1788 | 540 | 1167 |
| 4 dpi vs. control | | 27 | 2 | 0 | 2 | 3146 | 191 | 339 | 837 |
| 7 dpi vs. control | | 51 | 1 | 1 | 1 | 3122 | 0 | 42 | 215 |
| 10 dpi vs. control | | 64 | 0 | 2 | 6 | 3108 | 0 | 41 | 179 |
| 16 dpi vs. control | | 6 | 0 | 0 | 0 | 3167 | 0 | 290 | 758 |
| Time series (1🡪16 dpi) | | 3068 | 1044 | 397 | 867 | 105 | 0 | 1 | 6 |

**S3 Table.** g:Profiler results of significantly enriched KEGG and Reactome pathways over time in Coho fin.

|  |  |  | **Enriched Timepoint (dpi)** | | | | |
| --- | --- | --- | --- | --- | --- | --- | --- |
| **Term** | **Description** | **Genes** | **1** | **4** | **7** | **10** | **16** |
| KEGG:04932 | Non-alcoholic fatty liver disease | ITCH,UQCRQ,EIF2S1,NDUFA5,COX4I1,CYCS,COX7A2,CASP3,CXCL8,NDUFA1,IL1B,COX8A,COX7B |  |  |  |  |  |
| KEGG:05152 | Tuberculosis | CLEC4E,KSR1,JAK1,MRC1,PPP3CC,TNF,CD209,IL12B,IL1B,IFNGR1,CLEC4M,FCER1G,JAK2,CASP3,CEBPB,BCL10,ITGAX,CYCS,BAX,BID,CTSS,CARD9,RAB5C,FCGR1A,SYK,RIPK2,TGFB1,IL10RB,CORO1A,TNFRSF1A,ITGB2,CALM3,MAPK9,CASP10,HSPD1,RAB7A,CTSD,NFYB,CASP8,ATP6V0C,MAPK13,MYD88,HSPA9 |  |  |  |  |  |
| REAC:R-HSA-6783783 | Interleukin-10 signaling | FCER2,CCL4,IL12B,CXCL8,IL1B,IL1R2,IL18,CCL2,PTGS2,ICAM1,TNFRSF1A,TNFRSF1B,TNF,CCL19,JAK1,CCR1 |  |  |  |  |  |
| REAC:R-HSA-3299685 | Detoxification of Reactive Oxygen Species | PRDX1,CYCS,GPX2,TXN,NCF2,NCF1,SOD3,CYBA,CAT |  |  |  |  |  |
| REAC:R-HSA-5628897 | TP53 Regulates Metabolic Genes | GLS,PRDX1,COX4I1,CYCS,GPX2,COX16,COX8A,COX7B,TXN |  |  |  |  |  |
| REAC:R-HSA-381042 | PERK regulates gene expression | ATF6,EIF2S1,NFYA,CXCL8,CEBPB,CCL2 |  |  |  |  |  |
| KEGG:04668 | TNF signaling pathway | ITCH,PIK3R1,TNFRSF1B,TNF,PTGS2,MMP9,CCL2,IL1B,VEGFC,MAPK3,CASP3,CEBPB,VCAM1,SOCS3,MLKL,FADD,ICAM1 |  |  |  |  |  |
| KEGG:04933 | AGE-RAGE signaling pathway in diabetic complications | PIK3R1,CXCL8,TNF,VEGFA,CCL2,SERPINE1,IL1B,AGT,VEGFC,MAPK3,CASP3,VCAM1 |  |  |  |  |  |
| KEGG:05142 | Chagas disease | GNAI3,C1QB,PIK3R1,CXCL8,TNF,IL12B,IFNGR1,CCL2,SERPINE1,IL1B,MAPK3 |  |  |  |  |  |
| KEGG:04625 | C-type lectin receptor signaling pathway | KSR1,CLEC4E,PIK3R1,CD209,CLEC6A,TNF,PTGS2,IL12B,FCER1G,IL1B,MAPK3,AKT1,CLEC4M,EGR3,BCL10 |  |  |  |  |  |
| KEGG:04657 | IL-17 signaling pathway | MMP13,CXCL8,TNF,PTGS2,MMP9,CCL2,IL1B,MAPK3,CASP3,CEBPB,FOSL1 |  |  |  |  |  |
| KEGG:05133 | Pertussis | GNAI3,C1QB,CXCL8,TNF,IL12B,C4BPA,IL1B,MAPK3,CASP3 |  |  |  |  |  |
| KEGG:05418 | Fluid shear stress and atherosclerosis | RAC2,PIK3R1,IL1R2,TNF,MMP9,VEGFA,TXN,CCL2,NCF2,IL1B,PLAT,VCAM1,NCF1 |  |  |  |  |  |
| REAC:R-HSA-6785807 | Interleukin-4 and Interleukin-13 signaling | PIK3R1,FCER2,TNFRSF1B,CXCL8,TNF,PTGS2,MMP9,IL12B,VEGFA,CCL2,IL1B,VCAM1,BATF,SOCS3,IL13RA2,ICAM1,HSPA8,ITGAX,IL6R,JUNB,ALOX5,PIM1,HMOX1,SOCS1,JAK1,PTGS2,JAK2,CDKN1A,BCL2L1,ANXA1,IL2RG,ITGB2,HSP90AA1 |  |  |  |  |  |
| REAC:R-HSA-6798695 | Neutrophil degranulation | SPTAN1,CPNE3,CXCR1,TNFRSF1B,CD33,PIGR,FPR2,MMP9,CFD,ADAM8,FGL2,GPR84,C5AR1,CEACAM6,TMC6,P2RX1,PNP,GLIPR1,HVCN1,LYZ,GMFG,FCER1G,AP2A2,TIMP2,GSN,ARG1,ARHGAP45,ITGAX,LTA4H,PGAM1,HPSE,PSMB7,ALOX5,CD53,RHOF,CYBA,DEGS1,DBNL,DOCK2,SERPINB1,CRACR2A,DSP,CTSS,GPI,RAB5C,LTF,PYGL,MVP,NCKAP1L,RAB37,SIGLEC14,B4GALT1,RAB44,BIN2,CYBB,PTPRC,PKM,COTL1,CAB39,RHOG,UNC13D,STK10,ARL8A,QSOX1,DPP7,IRAG2,OLFM4,ITGB2,HSP90AA1,QPCT,MAGT1,GYG1,PADI2,CTSB,ATP11B,PSMD6,CD63,PSMD12,SURF4,CTSC,BPI,SNAP29,RAB7A,CTSD,PLAU,RAB6A,PSMD13,GALNS,ATP6AP2,ATP6V0C,ACTR2,TSPAN14,CAP1,IST1,PDAP1,ARSB,PSMC2,HEXB,CYFIP1,PA2G4,IDH1,EEF2,XRCC6,EEF1A1,PRDX6,PRDX4,STOM,PAFAH1B2 |  |  |  |  |  |
| REAC:R-HSA-9018678 | Biosynthesis of specialized proresolving mediators (SPMs) | PTGR1,PTGS2,ALOX5AP,LTA4H,ALOX5,EPHX2 |  |  |  |  |  |
| REAC:R-HSA-2682334 | EPH-Ephrin signaling | MYH9,ADAM10 |  |  |  |  |  |
| KEGG:05130 | Pathogenic Escherichia coli infection | EZR,MYH9,MYO1E |  |  |  |  |  |
| KEGG:05145 | Toxoplasmosis | JAK1,GNAI3,TNF,IL12B,LAMC2,PIK3R5,IFNGR1,JAK2,CASP3,LAMB3,LAMA3,ITGA6,CYCS,ALOX5,BCL2L1,PIK3CG,SOCS1,TGFB1,IL10RB,MAP3K7,TNFRSF1A,GNAI2,MAPK9,PDPK1,CASP8,MAPK13,MYD88,GNAI1,AKT1,STAT1,NFKBIA,NFKBIB,GNAO1,MAPK12,LAMB4,XIAP |  |  |  |  |  |
| REAC:R-HSA-449147 | Signaling by Interleukins | JAK1,CISH,FCER2,IL21R,TNFRSF1B,HNRNPA2B1,IL1R2,TNF,MMP9,PTGS2,CCL4,CSF2RB,CXCL8,BATF,IL12B,PSMA4,CCL19,IL1B,MAPKAPK3,LIFR,VCAM1,CCR1,JAK2,CASP3,DUSP7,SOCS3,ITGAX,YWHAZ,PSMB7,HCK,TEC,CDKN1A,ALOX5,ICAM1,HIF1A,FYN,CA1,IL6R,BCL2L1,LCP1,INPP5D,PTK2B,CSF3R,IL31RA,MSN,PIM1,LYN,SYK,RIPK2,IL7R,CDC42,SOCS1,CSF2RA,JUNB,IL17RC,PTPN18,CSF1R,ANXA1,IL10RB,MAP3K7,IL13RA1,MAP2K4,IL2RG,TNFRSF1A,ITGB2,HSP90AA1,CD4,PTPN9,MAPK9,STAT4,VRK3,IL13RA2,PSMD6,PSMD12,CUL1,PSMD13,VAMP7,MYD88,HSPA9,GRB2,STXBP2,IL12RB2,PSMA7,PELI2,PSMC1,PSMD5,PSMC2,UBE2N,AKT1 |  |  |  |  |  |
| REAC:R-HSA-168249 | Innate Immune System | MYH9,CLEC4E,TNFAIP3,SPTAN1,CPNE3,RAC2,ITCH,ATP6V1A,CXCR1,TNFRSF1B,CD300LB,CD33,PIGR,CFH,FPR2,MMP9,CD209,CFD,C4BPA,PSMA4,ADAM8,NLRC3,FGL2,IL1B,GPR84,C5AR1,TXN,MAPKAPK3,CEACAM6,TMC6,P2RX1,PNP,NCF1,GLIPR1,HVCN1,LYZ,NCF2,GMFG,FCER1G,AP2A2,TIMP2,GSN,BCL10,ARG1,ARHGAP45,DUSP7,LAT2,MUC5AC,ITGAX,LTA4H,TRIM25,PGAM1,CD46,HPSE,PSMB7,HCK,TEC,ALOX5,CD53,RHOF,FYN,CYBA,C6,DEGS1,DBNL,BCL2L1,DOCK2,SERPINB1,CRACR2A,DSP,CTSS,RASGRP2,GPI,CARD9,RAB5C,LTF,PYGL,FCGR1A,MVP,WAS,NCKAP1L,RAB37,SIGLEC14,B4GALT1,PSTPIP1,RAB44,LYN,SYK,BIN2,RIPK2,CDC42,CYBB,SOCS1,PTPRC,PKM,DEFA6,COTL1,CAB39,PRKCQ,MAP3K7,PTK2,RHOG,UNC13D,MAP2K4,STK10,ARL8A,QSOX1,DPP7,IRAG2,OLFM4,ITGB2,HSP90AA1,QPCT,UBE2D2,ITPR3,CD4,CALM3,MAGT1,ABI1,MAPK9,GYG1,CASP10,PADI2,PLCG2,VRK3,CTSB,ATP11B,PSMD6,CD63,PSMD12,SURF4,CTSC,ELMO1,BPI,SNAP29,LGMN,RAB7A,CTSD,ACTB,CUL1,PLAU,RAB6A,PSMD13,GALNS,ATP6AP2,PDPK1,CTSV,CASP8,ATP6V0C,ACTR2,MAPK13,MYD88,TSPAN14,ACTR3,CAP1,PSMA7,PELI2,IST1,ATP6V1H,PDAP1,PSMC1,PSMD5,ARSB,PRKCSH,ACTG1,PSMC2,UBE2N,HEXB,CYFIP1,PA2G4,IDH1 |  |  |  |  |  |
| REAC:R-HSA-168256 | Immune System | MYH9,CLEC4E,KSR1,JAK1,RNF19A,CLTC,TNFAIP3,SPTAN1,RASAL2,CPNE3,RAC2,CD22,MRC1,ITCH,CISH,FCER2,ATP6V1A,TNFSF13B,CXCR1,NRG2,IL21R,TNFRSF1B,HNRNPA2B1,FGF10,CD300LB,DLG1,CD33,PIGR,CD300LF,SIGLEC10,IL1R2,PJA2,CFH,TNF,FPR2,IRF1,MMP9,PTGS2,CCL4,CSF2RB,CD209,CFD,TNFRSF6B,C4BPA,CXCL8,BATF,ZNRF1,IL12B,EPGN,PSMA4,ADAM8,NLRC3,TUBB2A,FGL2,CCL19,CD274,IL1B,GPR84,MET,C5AR1,TXN,MAPKAPK3,CEACAM6,LIFR,IFNGR1,VCAM1,TMC6,P2RX1,PNP,SIGLEC1,NCF1,GLIPR1,HVCN1,CCR1,LYZ,NCF2,GMFG,FCER1G,AP2A2,JAK2,CDH1,CASP3,TIMP2,GSN,HBEGF,BCL10,ARG1,ARHGAP45,DUSP7,SOCS3,LAT2,MUC5AC,ITGAX,LTA4H,TRIM25,PGAM1,CD46,SAR1B,DUSP5,HPSE,DUSP1,YWHAZ,PSMB7,HCK,TEC,CDKN1A,IFI30,ALOX5,CD53,ICAM1,SIGLEC11,RHOF,HIF1A,FYN,CYBA,FYB1,C6,CA1,DEGS1,DBNL,IL6R,BCL2L1,DOCK2,SERPINB1,ITGB3,CRACR2A,LCP1,RAP1GAP,DSP,CTSS,RASGRP2,INPP5D,GPI,FBXL5,CARD9,RAB5C,LTF,PYGL,PTK2B,FCGR1A,MVP,WAS,CSF3R,IL31RA,NCKAP1L,MSN,PIM1,RAB37,SIGLEC14,B4GALT1,PSTPIP1,RAB44,IRF5,LYN,NFKBIE,SYK,BIN2,RIPK2,AP1G1,IL7R,CDC42,EIF4E,CYBB,SOCS1,CSF2RA,PTPRC,PKM,JUNB,DEFA6,RNF126,VASP,COTL1,IL17RC,CAB39,PTPN18,TRIM69,CSF1R,ANXA1,PRKCQ,IL10RB,KIT,CD226,MAP3K7,AP1M2,KPNA3,PTK2,IL13RA1,RHOG,UNC13D,MAP2K4,IRF2,IL2RG,STK10,ARL8A,QSOX1,DPP7,IRAG2,TNFRSF1A,OLFM4,ITGB2,HSP90AA1,QPCT,UBE2C,UBE2D2,ITPR3,NEDD4L,CD4,PTPN9,CALM3,MAGT1,ABI1,MAPK9,GYG1,CASP10,PADI2,STAT4,PLCG2,RAE1,VRK3,CTSB,IL13RA2,ATP11B,SMURF2,PSMD6,KPNA2,FBXO6,EREG,CD63,PSMD12,SURF4,RACGAP1,CTSC,ELMO1,BLMH,PPP2R5A,BPI,SNAP29,LGMN,CDC20,RAB7A,CTSD,UBA5,HACE1,TUBB3,ACTB,THOP1,YWHAB,NUP35,CUL1,PLAU,RAB6A,UBE2G1,CALR,PSMD13,GALNS,UBE2E1,ATP6AP2,PDPK1,VAMP7,CTSV,CASP8,ATP6V0C,ACTR2,MAPK13,MYD88,AP1S1,TSPAN14,DYNC1LI2,ERBB2,CLTA,HSPA9,GRB2,MAP2K2,STXBP2,EIF4A1,IL12RB2,ACTR3,CAP1,ABCE1,PSMA7,PELI2,PDIA3,IST1,ATP6V1H,PDAP1,PSMC1,PSMD5,DCTN2,ARSB,PRKCSH,ACTG1,PSMC2,TUBA3D,UBE2N,HEXB,KPNA5,DYNC1I2,CYFIP1,PA2G4,AKT1,CSK,IDH1 |  |  |  |  |  |
| REAC:R-HSA-2142753 | Arachidonic acid metabolism | PTGS2,GPX2,ALOX5AP,ALOXE3,CYP4F2,LTA4H,PTGES,GGT5,ALOX5,GGT1,EPHX2,GPX4,CYP4B1,PTGR1,PTGS1 |  |  |  |  |  |
| REAC:R-HSA-1280215 | Cytokine Signaling in Immune system | KSR1,JAK1,SPTAN1,RASAL2,CISH,FCER2,TNFSF13B,NRG2,IL21R,TNFRSF1B,HNRNPA2B1,FGF10,DLG1,IL1R2,TNF,IRF1,MMP9,PTGS2,CCL4,CSF2RB,TNFRSF6B,CXCL8,BATF,IL12B,EPGN,PSMA4,CCL19,IL1B,MET,MAPKAPK3,LIFR,IFNGR1,VCAM1,CCR1,JAK2,CASP3,HBEGF,DUSP7,SOCS3,ITGAX,TRIM25,DUSP5,DUSP1,YWHAZ,PSMB7,HCK,TEC,CDKN1A,IFI30,ALOX5,ICAM1,HIF1A,FYN,CA1,IL6R,BCL2L1,ITGB3,LCP1,INPP5D,PTK2B,FCGR1A,CSF3R,IL31RA,MSN,PIM1,IRF5,LYN,SYK,RIPK2,IL7R,CDC42,EIF4E,SOCS1,CSF2RA,JUNB,IL17RC,PTPN18,CSF1R,ANXA1,IL10RB,KIT,MAP3K7,KPNA3,PTK2,IL13RA1,MAP2K4,IRF2,IL2RG,TNFRSF1A,ITGB2,HSP90AA1,CD4,PTPN9,CALM3,MAPK9,STAT4,RAE1,VRK3,IL13RA2,PSMD6,KPNA2,EREG,PSMD12,PPP2R5A,ACTB,YWHAB,NUP35,CUL1,PSMD13,UBE2E1,PDPK1,VAMP7,MYD88,ERBB2,HSPA9,GRB2,MAP2K2,STXBP2,EIF4A1,IL12RB2,ABCE1,PSMA7,PELI2,PSMC1,PSMD5,ACTG1,PSMC2,UBE2N,KPNA5,AKT1,CSK |  |  |  |  |  |
| REAC:R-HSA-2142691 | Synthesis of Leukotrienes (LT) and Eoxins (EX) | ALOX5AP,CYP4F2,LTA4H,GGT5,ALOX5,GGT1,CYP4B1,PTGR1 |  |  |  |  |  |
| REAC:R-HSA-446107 | Type I hemidesmosome assembly | LAMC2,LAMB3,LAMA3,ITGA6,CD151,PLEC,COL17A1,DST |  |  |  |  |  |
| KEGG:04640 | Hematopoietic cell lineage | CD22,FCER2,ITGA3,ITGA2,IL1R2,IL1B,CD33,CD44,ITGA6,IL6R,FCGR1A,CSF3R,IL7R,ITGB3,ITGA4,CSF1R |  |  |  |  |  |
| REAC:R-HSA-1222556 | ROS and RNS production in phagocytes | ATP6V1C1,HVCN1,RAC2,ATP6V1A,NCF1,NCF2,MPO,CYBB,CYBA,ATP6V0D1 |  |  |  |  |  |
| REAC:R-HSA-1474244 | Extracellular matrix organization | ADAM10,COL15A1,FBN1,MMP13,CAST,MMP9,ITGA3,ADAM8,ITGA2,LAMC2,SERPINE1,MMP17,LAMA3,CEACAM6,CD44,ITGAX,ITGA6,LAMB3,CDH1,TIMP2,VCAM1,THBS1 |  |  |  |  |  |
| REAC:R-HSA-1474228 | Degradation of the extracellular matrix | ADAM10,COL15A1,FBN1,MMP13,CAST,MMP9,ADAM8,LAMC2,MMP17,LAMA3,CD44,LAMB3,CDH1,TIMP2 |  |  |  |  |  |
| REAC:R-HSA-351906 | Apoptotic cleavage of cell adhesion proteins | DSG2,CDH1,CASP3,TJP2,OCLN,PKP1 |  |  |  |  |  |
| REAC:R-HSA-210991 | Basigin interactions | SLC7A11,SLC16A3,ITGA3,MAG,ITGA6,SLC7A5,SLC3A2 |  |  |  |  |  |
| REAC:R-HSA-9018678 | Biosynthesis of specialized proresolving mediators (SPMs) | PTGR1,PTGS2,ALOX5AP,LTA4H,ALOX5,EPHX2 |  |  |  |  |  |
| REAC:R-HSA-114604 | GPVI-mediated activation cascade | RAC2,FCER1G,FYN,PIK3R5,LYN,PIK3CG,SYK,PTPN6,RHOG |  |  |  |  |  |
| REAC:R-HSA-111465 | Apoptotic cleavage of cellular proteins | ACIN1,DSG2,CDH1,GSN,CASP3,CASP6,DBNL,TJP2,OCLN,PKP1,PLEC,SPTAN1 |  |  |  |  |  |
| REAC:R-HSA-210990 | PECAM1 interactions | FYN,LYN,INPP5D,ITGB3,PTPN6 |  |  |  |  |  |
| KEGG:04640 | Hematopoietic cell lineage | CD22,FCER2,ITGA3,ITGA2,IL1R2,IL1B,CD33,CD44,ITGA6,IL6R,FCGR1A,CSF3R,IL7R,ITGB3,ITGA4,CSF1R |  |  |  |  |  |
| KEGG:04144 | Endocytosis | EHD4,ITCH,RABEP1,PIP5KL1,WASHC2A |  |  |  |  |  |
| REAC:R-HSA-5621480 | Dectin-2 family | CLEC4E,MUC5AC,FCER1G,LYN |  |  |  |  |  |

**S1 File. DETs in *Okis* and *Lsal*.**

**S2 File. RT-qPCR validation of select transcripts overexpressed in the *Okis* transcriptome.**

**S3 File. Overexpressed genes in the *Okis* transcriptome grouped by coincident *Lsa*l-reads.**

**S4 File. Manually annotated *Okis* transcriptome.**
